# Global Developmental Gene Programing Involves a Nuclear Form of Fibroblast Growth Factor Receptor-1 (FGFR1)

**DOI:** 10.1371/journal.pone.0123380

**Published:** 2015-04-29

**Authors:** Christopher Terranova, Sridhar T. Narla, Yu-Wei Lee, Jonathan Bard, Abhirath Parikh, Ewa K. Stachowiak, Emmanuel S. Tzanakakis, Michael J. Buck, Barbara Birkaya, Michal K. Stachowiak

**Affiliations:** 1 Department of Pathology and Anatomical Sciences, Western New York Stem Cell Culture and Analysis Center, State University of New York at Buffalo, Buffalo, New York, United States of America; 2 Next-Generation Sequencing and Expression Analysis Core, State University of New York at Buffalo, Buffalo, New York, United States of America; 3 Department of Chemical and Biological Engineering, Western New York Stem Cell Culture and Analysis Center, State University of New York at Buffalo, Buffalo, New York, United States of America; 4 Department of Biochemistry, Genomics and Bioinformatics Core, Western New York Stem Cell Culture and Analysis Center, State University of New York at Buffalo, Buffalo, New York, United States of America; University of Massachusetts Medical, UNITED STATES

## Abstract

Genetic studies have placed the *Fgfr1* gene at the top of major ontogenic pathways that enable gastrulation, tissue development and organogenesis. Using genome-wide sequencing and loss and gain of function experiments the present investigation reveals a mechanism that underlies global and direct gene regulation by the nuclear form of FGFR1, ensuring that pluripotent Embryonic Stem Cells differentiate into Neuronal Cells in response to Retinoic Acid. Nuclear FGFR1, both alone and with its partner nuclear receptors RXR and Nur77, targets thousands of active genes and controls the expression of pluripotency, homeobox, neuronal and mesodermal genes. Nuclear FGFR1 targets genes in developmental pathways represented by Wnt/β-catenin, CREB, BMP, the cell cycle and cancer-related TP53 pathway, neuroectodermal and mesodermal programing networks, axonal growth and synaptic plasticity pathways. Nuclear FGFR1 targets the consensus sequences of transcription factors known to engage CREB-binding protein, a common coregulator of transcription and established binding partner of nuclear FGFR1. This investigation reveals the role of nuclear FGFR1 as a global genomic programmer of cell, neural and muscle development.

## Introduction

Development of a multicellular organism from a single cell is regulated by myriads of TFs and requires the coordinated regulation of multi-gene programs. The "Integrative Nuclear Fibroblast Growth Factor Receptor-1 (FGFR1) Signaling" (INFS) pathway has been shown to mediate cellular development and differentiation programs activated by numerous signals [1, 2]. At the center of the INFS module are proteins that bear the name FGF for historic reasons. Neither FGFs nor FGFRs exist in single-cell organisms, but are common to eumetazoans and essential for the generation of tissues with specialized cells [1]. Mutations of the *Fgfr1* gene interfere with gastrulation, as well as with development of the neural plate and neural crest, central nervous system, and somites by affecting the expression of diverse genes [3–6] and microRNAs [7, 8] that control development. These observations firmly place *Fgfr1* at the top of the developmental hierarchy.

The *C*. *elegans* [9] FGF ortholog LET-756 contains 3 nuclear localization signal (NLS) peptides, and its biological effects depend on its nuclear accumulation. During evolution of the mammalian FGF family, some members retained an NLS, and/or acquired a cleavable secretion signal peptide (SP). NLS-containing FGFs, e.g., the 23 kDa FGF-2, act in the nucleus to promote differentiation, whereas secreted members of the FGF family, e.g., 18 kDa FGF-2, act on the cell surface and serve a mitogenic function [10–13]. Individual FGFRs (in mammals, FGFR1-4) likewise have adaptations that direct them to other cellular compartments [14]. For example, an atypical transmembrane domain in FGFR1 allows the newly translated receptor to be released from the pre-Golgi membrane and to translocate into the nucleus, a process facilitated by its FGF-2 ligand and importin-β[15]. The accumulation of hypoglycosylated nuclear FGFR1 (nFGFR1) is stimulated by a variety of developmental signals, including various growth factors, hormones, and neurotransmitters as well as a reduction in cell contact. This is the reason that this pathway is referred to as integrative [1, 15].

The INFS mechanism is involved primarily in developmental transitions, most commonly the switches to differentiation and post-mitotic development [1, 10]. Transfection of the recombinant, constitutively nuclear variant FGFR1(SP-/NLS), in which the cleavable SP is replaced with the NLS of FGF2, and of dominant-negative variant FGFR1(SP-/NLS)(TK-), which lacks the tyrosine kinase (TK) domain, showed that nFGFR1 is sufficient and necessary for neuronal differentiation, both in the mouse brain [16, 17] and in cultured cells treated with NGF, BMP, or cAMP [18–21]. Once in the nucleus, FGFR1 directly binds and activates CREB Binding Protein (CBP), a histone acetyltransferase and coactivator of multiple transcription factors (TFs). Through this interaction, nFGFR1 binds to cAMP-response elements (CREs) and activator protein-1 (AP-1) sites within active neuronal genes, and augments sequence-specific elements regulated by CBP, including CRE, AP-1, and NF-κB [20]. In addition, recent studies have demonstrated that both full-length and truncated forms of FGFR1 accumulate in cancer cells and thereby promote metastasis [22–24].

Within mouse Embryonic Stem Cells (ESCs), core networks of interconnected TFs control the ability of these cells to maintain the pluripotent state or differentiate into lineages of all three germ layers [25, 26]. Retinoic Acid (RA) has broad regulatory functions during embryonic development [27], triggering transcription cascades that cause ESCs to differentiate into neuronal, cardiac, myogenic, adipogenic, and vascular smooth muscle cells, with the exact outcome depending on ligand concentration. At high concentrations [1–10 μM], RA promotes exit from the pluripotent state and development specifically along the neuronal lineage, while also inhibiting glial-cell development [28–31]. Within a few hours, ESCs exit the cell cycle and upregulate neurogenic and neuronal genes, and by 48h, the cells display a neuronal morphology (including long neurites and growth-cone endings), and express neuron-specific -III tubulin, MAP2, neurofilament L, tyrosine hydroxylase (TH), and glutamate and acetylcholine receptors [28–31]. RA signaling is mediated by both retinoic acid receptors (RARs) and retinoid X receptors (RXRs), which can act as homo or heterodimers on RA-responsive elements (RARE) within RA-regulated genes [32]. Additionally, RXR is highly versatile with respect to its heterodimerization; among the many other nuclear receptors with which it can interact are two members of the orphan nuclear subfamily, Nur77 and Nurr1. These factors also function independently by binding Nur-response elements, as monomers (NBRE) and dimers (NurRE) [32–34]. Recent studies in our laboratory have shown that nuclear accumulation of FGFR1 is a common response to RA in human ESCs and mESCs, and that once in the nucleus this receptor forms complexes with RXR, RAR and Nurs. These complexes bind to RARE, NBRE and NurRE-like sequences within RA-activated *Fgfr1*, *Fgf-2* and *Th* genes, and synergistically activate isolated RA- and Nur-responsive elements [21, 28, 35]. Furthermore, loss- and gain-of-function experiments of nFGFR1 have demonstrated that this protein is necessary for RA-induced differentiation of neurons, and that it is sufficient to induce differentiation in the absence of RA stimulation [28].

How can a single nuclear protein program the development of ESCs—a process that involves the coordinated regulation of thousands of genes that are located on different chromosomes and contain diverse regulatory elements? Given that nFGFR1 binds to and activates CBP [20], a histone acetyltransferase and coactivator of multiple TFs, it could potentially act as a global master regulator that delivers the RA signal to a variety of genes, including some that lack an RXR or Nur-related site. Therefore, we hypothesized that nFGFR1 mediates RA-induced programming of ESCs to a neural fate by targeting selected “master developmental” genes and/or interacting directly with multiple sets of genes within diverse development pathways. We tested this hypothesis by performing chromatin immunoprecipitation (ChIP) and mRNA sequencing, identifying networks of genes that are influenced by FGFR1, RXR and Nur77 binding and characterizing the associated gene regulation during RA-induced neuronal differentiation of ESCs.

## Results

### FGFR1, RXR and Nur77 bind to chromatin sites throughout the genome

**Fig 1:**
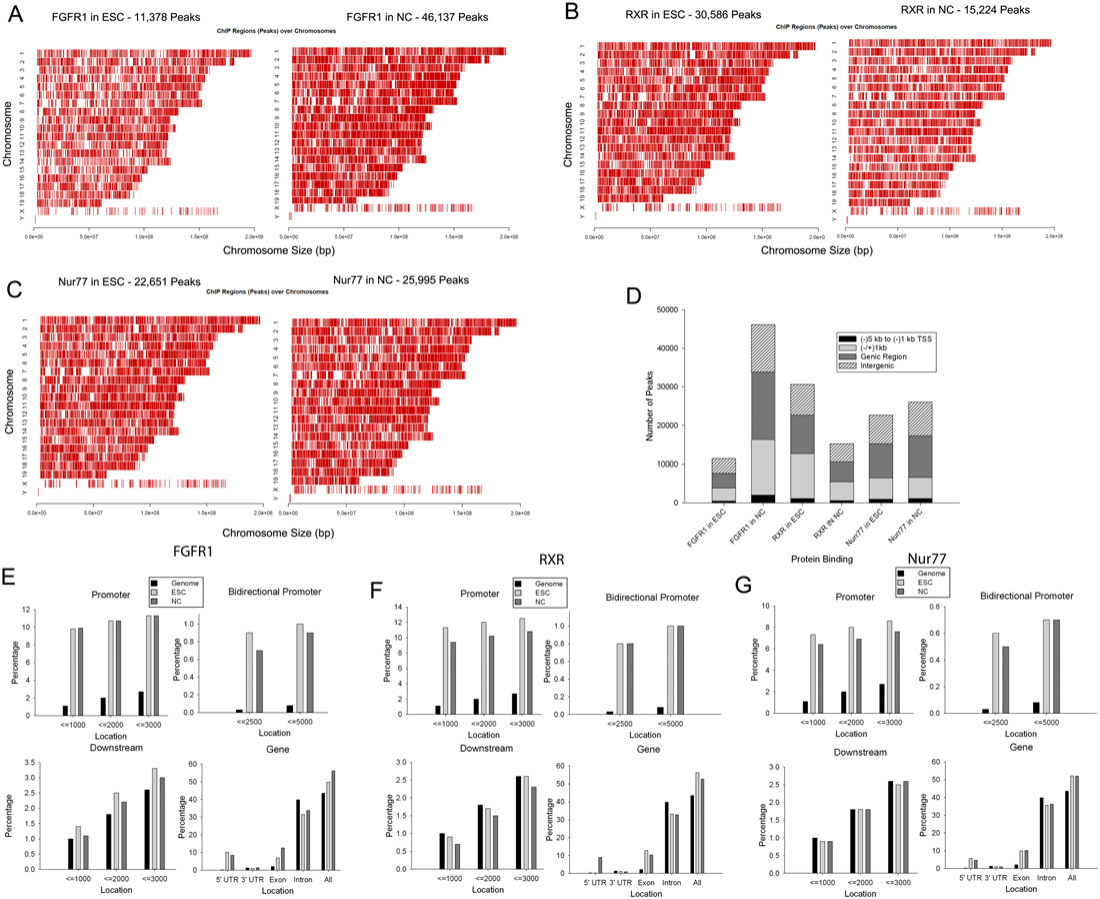
Genome-wide analyses of nFGFR1, RXR and Nur77 binding in pluripotent ESCs and RA-induced NCs. **(A)** nFGFR1, **(B)** RXR and **(C)** Nur77 peaks are present on all chromosomes, in both ESCs and NCs. **(D)** Genomic distribution of nFGFR1, RXR and Nur77 peaks within proximal promoters (-1kb to +1 kb relative to TSS), distal promoters (-5 kb to -1 kb relative to TSS), genic and intergenic regions in ESCs and NCs. **(E-G)** Enrichment of FGFR1, RXR and Nur77 peaks within promoter and genic regions.

To identify the chromatin-binding patterns of nFGFR1, RXR and Nur77, we performed ChIP-seq on extracts from pluripotent ESCs maintained in the presence of leukemia inhibitory factor (LIF), essential for maintaining stem cell pluripotency, and from LIF-free monolayers treated with 1 μM RA for 2 days to induce Neuronal Cell (NC) differentiation. In both ESCs and NCs, the peaks of nFGFR1, RXR and Nur77 binding were heterogeneously distributed across the chromosomes (Fig 1A–1C and S1A—S1C Fig). Specifically, in the NCs we observed: a four-fold increase in the number of nFGFR1 binding sites (11,378 peaks in ESCs and 46,137 in NCs); a two-fold decrease in the number of RXR sites (30,586 peaks in ESCs and 15,224 in NCs); and no significant change in the number of Nur77 sites (22,651 peaks in ESCs and 25,995 in NCs) (S1 Table). Further analysis revealed that these global changes in binding reflected changes in all genomic regions, including: (i) the distal promoter (-5kb to -1kb TSS), (ii) the proximal promoter (-1kb to +1kb TSS), (iii) the gene body (+1kb TSS to 3’UTR), and (iv) intergenic regions (Fig 1D). Nevertheless, in both ESCs and NCs, the binding of all three factors was highly enriched within the upstream proximal promoter (-1kb), the bidirectional promoter and the 5’UTR, but not in the downstream promoter (+1kb), the 3’UTR, or introns (Fig 1E–1G). A comparison of sequences across vertebrate species revealed a high level of evolutionary conservation for sets of nFGFR1, RXR and Nur77 peaks, corroborating their importance as genomic regulators (S1D—S1F Fig). S1G Fig shows genome-browser views of binding by nFGFR1, RXR and Nur77 to previously identified target genes [28, 36].

### nFGFR1 binds to the genome independently and together with RXR and Nur77

**Fig 2:**
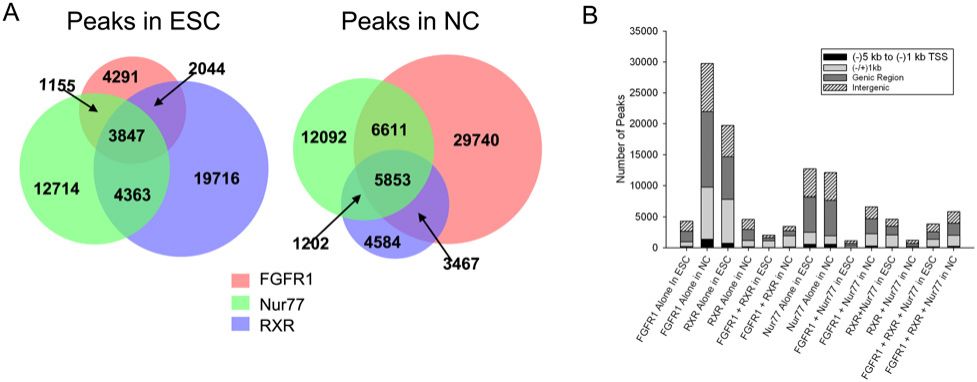
Genome-wide colocalization of nFGFR1, RXR and Nur77 peaks. **(A)** Venn diagram illustrates the number of individual and overlapping nFGFR1, RXR and Nur77 binding sites. **(B)** nFGFR1, RXR and Nur77 peaks colocalize within all genomic regions. Specifically in the proximal promoter and NCs, the number of sites at which RXR or Nur77 were bound together with nFGFR1 was markedly higher than the number of sites at which RXR or Nur77 were bound without nFGFR1.

To determine whether DNA nFGFR1 targeting of any part of the genome is dependent on co-targeting with RXR and/or Nur77 [21, 28], we analyzed genomic locations in which binding overlapped by a minimum of 1 base-pair (bp). In ESCs, 19% of all RXR and 22% of all Nur77 peaks colocalized with those for nFGFR1, and 45% of all sites co-occupied by RXR+Nur77 colocalized with nFGFR1 peaks. In NCs, the numbers were higher in all cases: the percentage of RXR peaks to which nFGFR1 colocalized was 61%, of Nur77 peaks to which nFGFR1 colocalized was 54%, and sites co-occupied by RXR+Nur77 that co-localized with nFGFR1 was 83% (Fig 2A). Thus, over the course of ESC differentiation into NCs, we observed an overall increase in the number of nFGFR1 sites bound by RXR and/or Nur77, in spite of the decrease of RXR and the small change in Nur77 binding. Most notably, as shown in Fig 2B, in all genomic regions we identified large numbers of sites to which nFGFR1 bound alone (37% in ESCs and 64% in NCs). Within the proximal and distal promoter regions, we observed prominent increases in the number of instances when nFGFR1 was bound, regardless of whether it was alone or colocalized with RXR or Nur77, but the number of RXR and RXR-Nur77 sites lacking nFGFR1 was low, and the number of sites bound by Nur77 alone remained relatively unchanged (Fig 2B). Thus, RA could potentially induce gene programing in differentiating NCs through an increase in nFGFR1 binding within regulatory regions of the mouse genome, either alone or together with RXR and Nur77.

### nFGFR1, RXR and Nur77 bind within promoters of active genes

**Fig 3:**
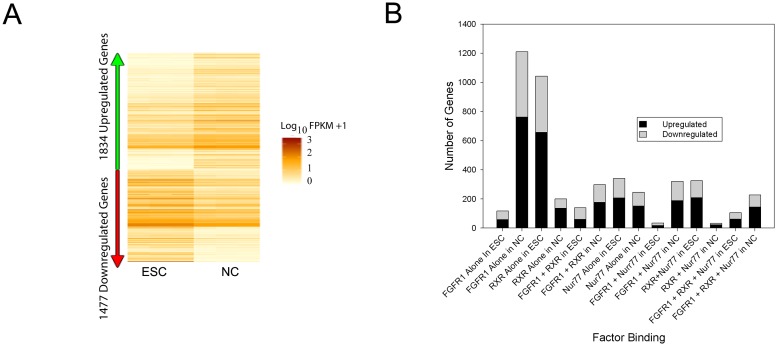
Binding of nFGFR1, RXR and Nur77 to expressed genes. **(A)** Heatmap representation of genes that were differentially expressed in pluripotent ESCs and RA-induced NCs from three independent biological replicates. Out of 14,443 expressed genes, 1,834 were up-regulated and 1,477 were down-regulated in NCs [Fold Change (FC) ≥-/+2.0 and p-value <0.035 were considered significant]. Values are displayed as fragments per kb of transcript per million fragments mapped (FPKM). **(B)** Binding of nFGFR1, RXR and Nur77 within the proximal promoter of differentially regulated genes. In NCs, the population of regulated genes that are targeted by nFGFR1 (2,058 genes) was markedly higher than the population of regulated genes that are not (480 genes).

To identify all mRNA genes expressed in both ESCs and NCs, we performed RNA-seq. In total, 14,443 expressed genes were detected, of which 1,834 were up-regulated and 1,477 down-regulated (fold change (FC) ≥-/+2.0 and p-value <0.035) during RA-induced neuronal differentiation (Fig 3A and S2 Table).

Combined analyses of the ChIP-seq and RNA-seq data sets revealed that >85% of the sites within the proximal promoter (-1kb to +1kb TSS) that were bound by nFGFR1, RXR, Nur77, individually or in combination, were associated with expressed genes (S2 Fig). Thus, all analyzed factors were targeted primarily to genes that are expressed as mRNAs in ESCs and NCs. The number of nFGFR1-targeted genes that were expressed (S2 Fig) and differentially regulated (Fig 3B) increased markedly during the transition from ESC to NC. Increases were also observed in the cases of genes whose promoters were co-targeted by nFGFR1 and RXR, Nur77 or both. In contrast, the population of genes bound by RXR and/or Nur77 but not nFGFR1, was markedly reduced. In NCs, the population of regulated genes that were targeted by nFGFR1 (2,058 genes) constituted over 62% of all differentially regulated genes; i.e., it was noticeably larger than the population of regulated genes that did not bind nFGFR1 (480 genes) (Fig 3B).

nFGFR1 targeted not only mRNA-encoding genes, but also expressed miRNA genes. Using miRNA-seq we identified 534 miRNAs that were expressed and 24 that were differentially regulated. In ESCs, nFGFR1 bound to the proximal promoters of only two expressed miRNAs. However, during RA-induced NC differentiation this number increased to 14 (S3 Table); among these were genes that promote neurogenesis [37].

One established marker of gene activation is histone variant H3.3 [38], which is incorporated into promoters containing nFGFR1 and Nur77 during RA-induced gene activation [28]. ChIP-seq with anti-H3.3 shows that, like nFGFR1 binding, such incorporation occurs globally. Moreover, the incorporation of H3.3 increased within nFGFR1 peaks within the promoters of expressed and differentially regulated genes (S3A—S3E Fig). These observations lend further support to the notion that nFGFR1 is a universal gene regulator.

### Pathways targeted by nFGFR1, RXR and Nur77 in ESCs and NCs are distinct

We next sought to identify biological pathways and networks in which the differentially expressed genes whose promoters are targeted by nFGFR1 are involved. As a first step, we utilized Ingenuity Pathway Analysis (IPA), an established tool for analyzing comprehensive genomic data.

**Fig 4:**
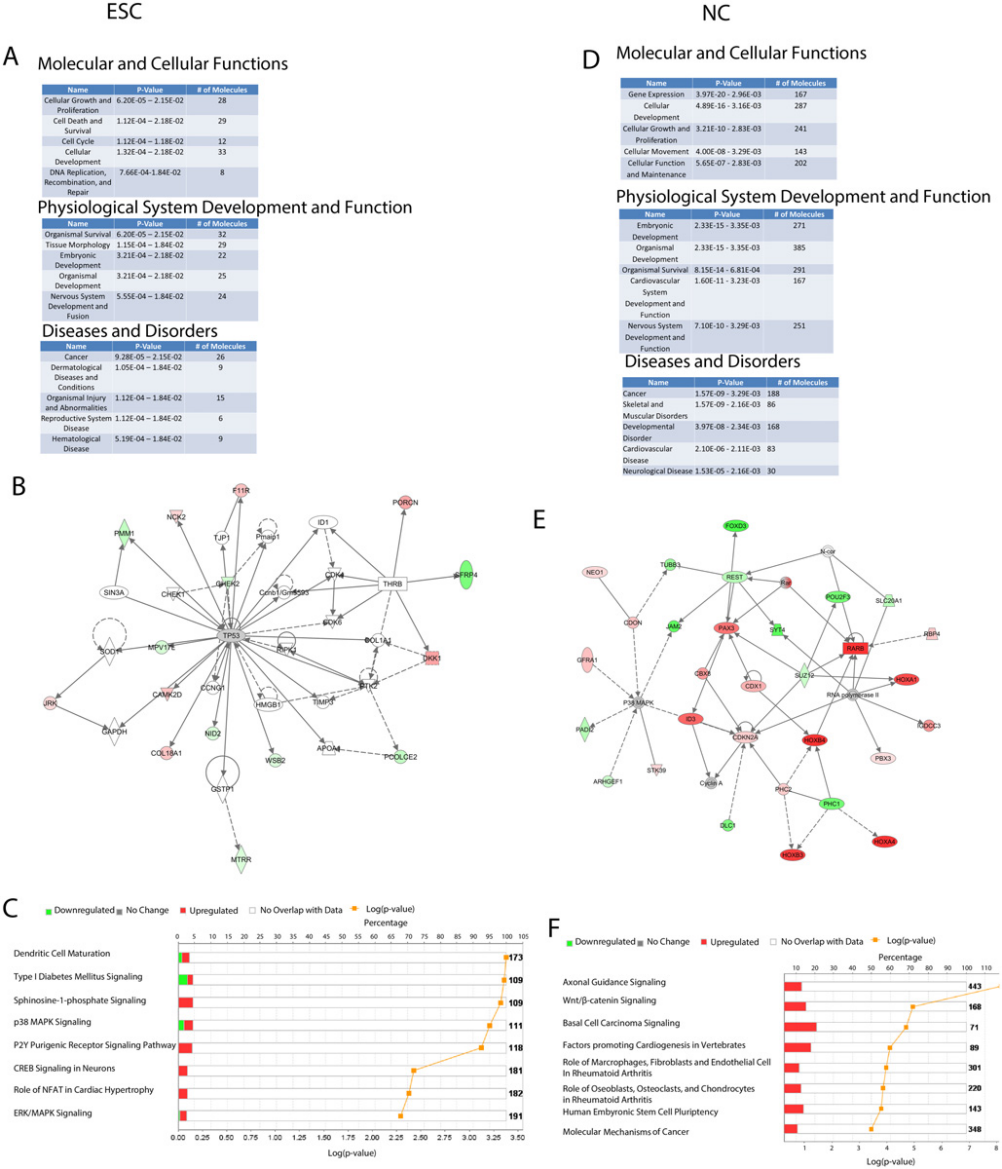
Ingenuity Pathway Analysis (IPA) of genes expressed differentially in ESCs and NCs and targeted by nFGFR1 only. Top biological functions, networks and diseases identified. Networks illustrate the degree of gene upregulation (red) and downregulation (green) by color intensity. Genes that were bound by nFGFR1 and not differentially regulated according to our cut-off are displayed in gray. Solid lines represent direct, and dotted lines indirect, interactions between genes in the network. A complete interpretation of network shapes and interactions can be found in Material and Methods. P-values were calculated using the right-tailed Fisher’s exact test. **(A-C)** nFGFR1 binding to genes in ESCs: **(A)** biological functions and diseases; **(B)** top network controlling cell proliferation and survival; and **(C)** eight top canonical pathways. **(D-F)** Binding of nFGFR1 to genes in in RA-differentiated NCs: (**D)** biological functions and diseases; **(E)** networks; and **(F)** eight top canonical pathways.

When the nFGFR1-targeted promoters not bound by RXR or Nur77 in pluripotent ESCs were analyzed, the top biological functions and diseases identified were cell cycle, growth and proliferation, development and cancer (Fig 4A). Within the top network, nFGFR1 targeted promoters of diverse genes including tumor protein 53 (*Tp53*), checkpoint kinase 1/2, *Dkk1* and *Camk2d* (Fig 4B and S4A Fig), all of which are known to control the cell cycle and are deregulated in various types of cancer [39–41]. Consistent with previous findings demonstrating that nFGFR1 mediates neuronal differentiation and gene activation through a CREB/CBP-dependent mechanism [19, 20, 42], one of the categories identified by our IPA analysis was “CREB signaling in neurons” (Fig 4C), in which nFGFR1 directly targets genes involved in CAMK, PI3K, MEK1/2, and adenylate cyclase signaling, all of which provide converging inputs promoting CREB activation (S4B Fig).

When nFGFR1 targeted promoters not bound by RXR or Nur77 in differentiated NCs were examined, the main functions identified were gene expression and various aspects of embryonic and nervous-system development (Fig 4D). In the nFGFR1-targeted developmental gene network (Fig 4E), the top down-regulated genes included the following: *Suz12*, a polycomb protein and direct repressor of RA-regulated genes [36, 43]; *Pouf3*, a regulator of the cell cycle; and *Foxd3* and *Rest*, master repressors of neural development [44, 45]. The top up-regulated genes targeted by nFGFR1 are critical for neural development (e.g., *Pax3* and *Irx3* [46, 47]), and some act in the RA-activated developmental pathway (e.g., various Homeobox genes [48], the *Rarb* receptor [36] and the RA-degradation enzyme *Cyp26a1* [36, 49]) (Fig 4E). Additional genes bound by nFGFR1 were found to be involved in axonal guidance (Fig 4F and S4C Fig) and Wnt/β-catenin signaling (diverse components including various Wnt ligands, the Frizzled 8 receptor, *Sox* factors, and *Porcn* (S4D Fig), a protein involved in Wnt biogenesis and recycling). These results are consistent with the established developmental roles of the *Fgfr1* gene [3, 21, 28], but go further—advancing a new model whereby the up-regulation of genes that promote neuronal development, and the down-regulation of genes that suppress this process, are mediated directly by nFGFR1.

In pluripotent ESCs, genes in which nFGFR1 targeted promoters also bound by RXR or Nur77 were organized into several canonical RXR- or Nur77-regulated pathways, for instance TGF- βsignaling [50], EGF and nitric oxide signaling [51], PPAR/RXR activation [51], tight-junction signaling [52], and histamine degradation [53] (S5A—S5B Fig). In NCs, the top pathways identified for genes bound by nFGFR1 and RXR were associated with various aspects of ESC pluripotency (S5C Fig), including BMPs and *Smad* genes (S5D Fig). Other pathways related to various aspects of embryonic and neuronal development, synaptic depression and dopamine signaling, with nFGFR1-Nur77 targeting dopamine receptor genes 2, 3, and 4 (S5E—S5F Fig). Importantly, many of these pathways were not identified within the gene networks targeted by nFGFR1 alone (Fig 4C, 4F and S5A—S5H Fig). These findings indicate that nFGFR1 has distinct effects when it targets the genome in the presence vs. absence of RXR and/or Nur77.

### nFGFR1 binds to and regulates genes of the pluripotency core

As our data indicated that nFGFR1 targets promoters of genes that are key to the RA-induced differentiation of ESCs, we sought confirmation of binding within these regions. To this end, we performed an additional ChIP-seq experiment in RA-induced NCs and a series of independent ChIP assays. The second ChIP-seq replicate confirmed that evolutionary conservation for nFGFR1-targeted sequences was high (S6A Fig). We identified 11,223 nFGFR1 binding sites within the proximal promoter region (S6B Fig), a number that did not differ significantly from the 14, 270 sites in the first experiment. Again, the density of nFGFR1 peaks was highest in the upstream promoter, bidirectional promoter, and 5’UTR, reaching 33-fold, 94-fold and 116-fold enrichment, respectively (S6C Fig).

**Fig 5:**
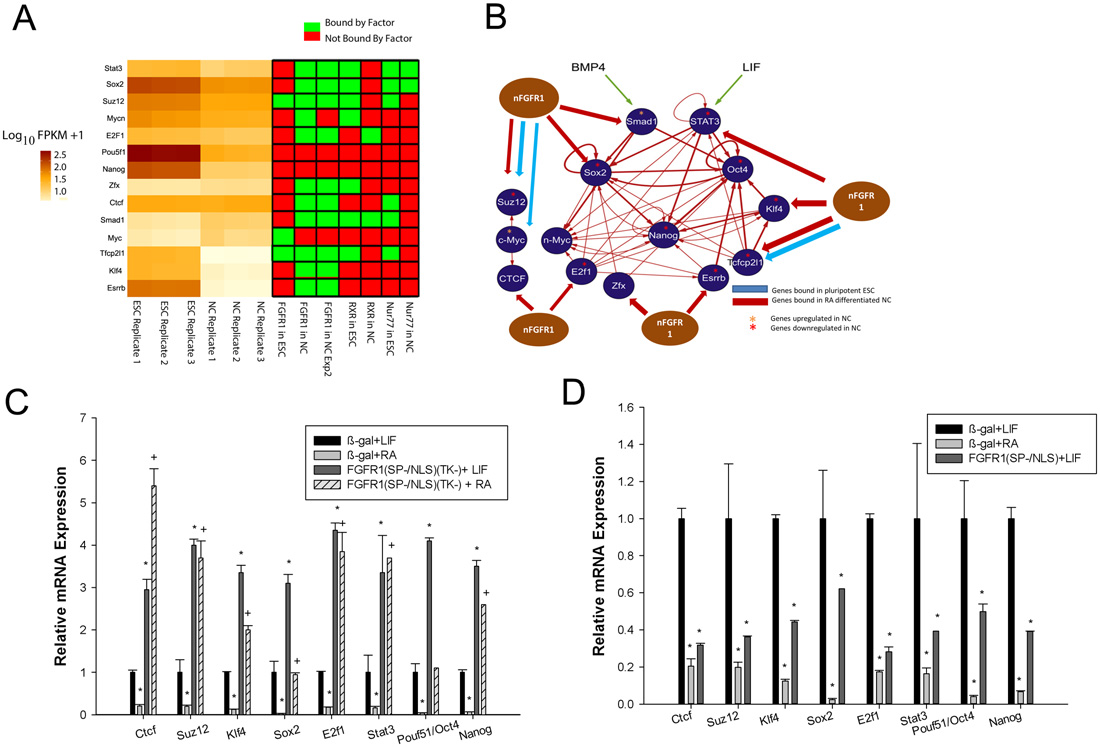
nFGFR1 targets pluripotentcy core genes and motifs in pluripotent transcription-factors (TFs). **(A)** Heatmap illustrating the expression patterns of core pluripotentcy genes (left) and associated proximal promoter binding of nFGFR1, RXR and Nur77 (right). **(B)** The pluripotent gene network is based on Chen et al.[25, 26]. The present investigation reveals core genes that have promoters targeted by nFGFR1 and were differentially regulated during the RA-induced transition from ESC to NC. **(C)** Expression of core pluripotentcy genes in the presence of dominant-negative nuclear FGFR1(SP-/NLS)(TK-). mRNA expression was measured in extracts from ESCs that had been transfected with either β-gal (control) or FGFR1(SP-/NLS)(TK-) and were then maintained in the presence of +LIF or +RA for 48 hours. In the presence of LIF, the block in nFGFR1 increased the basal expression of all genes examined. In RA-treated ESCs, the dominant-negative FGFR1 protein completely abolished the RA-induced downregulation of all members of the pluripotency core. p-value <0.05 * relative to β-gal+LIF; + relative to β-gal+RA. **(D)** Expression of core pluripotency genes in the presence of FGFR1(SP-/NLS). nFGFR1 in the presence of LIF repressed core pluripotency genes to an extent similar to RA alone. In the presence of LIF, transfection of FGFR1(SP-/NLS) induced the downregulation of all pluripotent genes. P-value <0.05 * relative to β-gal+LIF.

Although nFGFR1 had previously been shown to promote the neuronal differentiation of ESCs [28], whether it can also engage in maintenance of, or exit from, the pluripotent state was unknown. In both of our ChIP-seq experiments, nFGFR1 bound within promoters of the *Klf4*, *Sox2*, *Stat3*, *E2f1*, *Esrrb*, *Suz12*, *Smad1*, *Zfx*, *Tcfcp2l1*, and *Ctcf* genes, the majority of which were downregulated as ESCs differentiated to NCs (Fig 5A, 5B and S7A Fig). All of these nFGFR1-targeted genes belong to the pluripotency core transcriptional network described by Chen et al. [25]. The binding of nFGFR1 to selected pluripotency genes was confirmed in independent ChIP assays (S7B Fig). In pluripotent ESCs, nFGFR1 bound only within the promoters of *Suz12*, *Myc* and *Tcfcp2l1* (Fig 5A, 5B and S7A Fig). In contrast, RXR or Nur77 bound to nearly all pluripotency genes in these cells, and vacated these sites in NCs (Fig 5A, 5B and S7A Fig).

To determine whether nFGFR1 influences the expression of pluripotency genes, we modulated nFGFR1 function and measured levels of the candidate mRNA targets by real-time quantitative PCR (RT-qPCR). Since both the deletion and siRNA-mediated inhibition of the *Fgfr1* gene depletes both membrane-bound FGFR1 and nFGFR1, we used another strategy that has been established as effective in inhibiting nFGFR1 specifically, i.e., application of a dominant-negative receptor in which the tyrosine kinase (TK) domain is deleted (FGFR1(SP-/NLS) (TK-)). This exclusively nuclear protein prevents endogenous nFGFR1 from binding to targeted gene promoters [42] and the associated activation of target genes [19–21, 28, 35, 42, 54].

We analyzed the effects of FGFR1(SP-/NLS)(TK-) on genes that were down-regulated during NC differentiation and either bound (*Ctcf*, *Suz12*, *Klf4*, *Sox2*, *E2f1*, *and Stat3*) or not bound (*Oct4 and Nanog*) by endogenous nFGFR1. In ESCs, FGFR1(SP-/NLS)(TK-) led to a significant increase in the basal expression of all pluripotency genes examined (Fig 5C). This finding suggests that endogenous nFGFR1 regulates these genes both directly and indirectly. In β-galactosidase (β-gal)-transfected control cells, RA-treatment down-regulated the expression of all pluripotency genes examined (Fig 5C). The presence of FGFR1(SP-/NLS)(TK-) prevented or diminished down-regulation, and in many cases, the levels of the corresponding mRNA were higher than the basal levels in cells maintained in pluripotency (+LIF) medium (Fig 5C). Thus, endogenous nFGFR1 is necessary for the down-regulation of core pluripotency genes during RA-induced cell differentiation. FGFR1(TK-), a dominant-negative variant that acts on both the membrane-bound and nuclear forms of FGFR1, also increased basal expression of pluripotency genes (S7C Fig). However, its effect on the RA-induced down-regulation of nearly all genes was markedly diminished relative to that caused by FGFR1(SP-/NLS)(TK-) (Fig 5C); this may reflect the antagonistic functions of membrane-bound FGFR1 and nFGFR1 [19, 42].

To determine if nFGFR1 is sufficient to repress pluripotency genes as RA does, we transfected ESCs with constitutively active, nuclear FGFR1(SP-/NLS). This exclusively nuclear protein binds to CREs within promoters [42], activates transcription [15, 20, 21, 28, 35, 42] and promotes ESC differentiation in the absence of RA [28]. In the presence of LIF, FGFR1(SP-/NLS) led to a decrease in the expression of all pluripotency genes (Fig 5D). Its effect was comparable to that of RA treatment.

### nFGFR1 mediates RA activation of the Homeobox genes (3’ *Hoxa* cluster genes)

The *Hox* genes encode homeo-domain TFs, which are present in all metazoans and ensure both that the general body plan is established and that the central nervous system is organized [55]. In response to RA, *Hox* clusters are activated sequentially, according to chromosomal position (3’ to 5’) [48].

**Fig 6:**
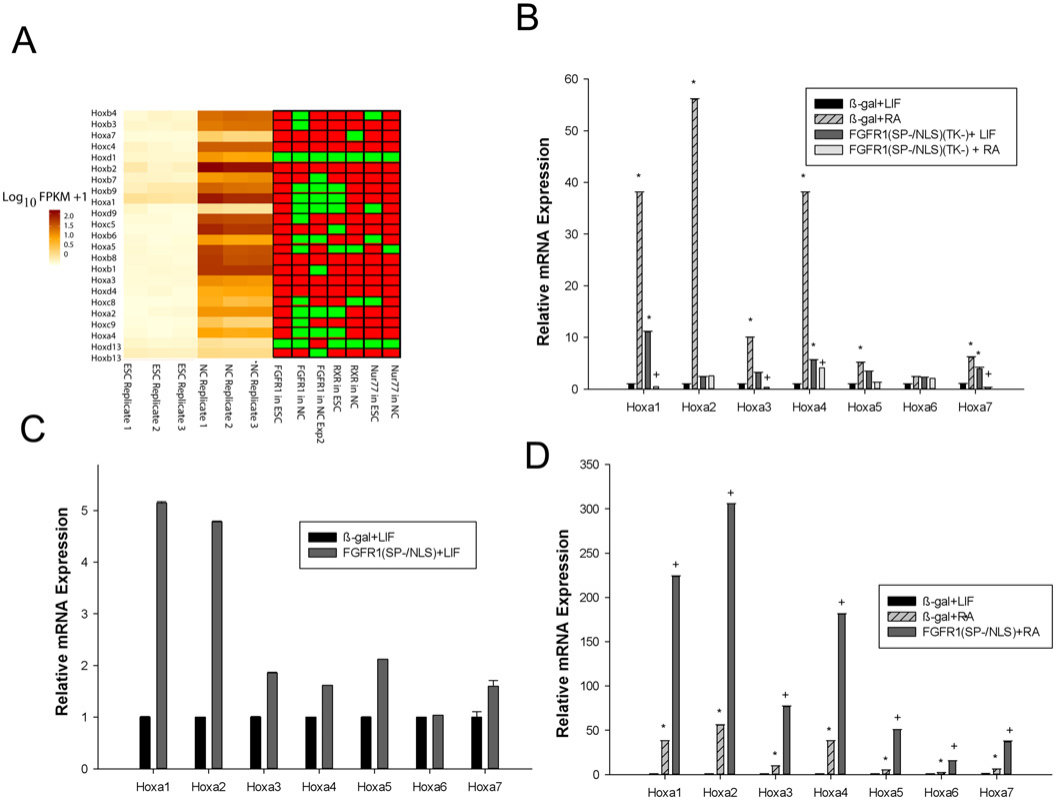
nFGFR1 regulates the expression of *Homeobox* genes in RA-treated NCs. **(A)** Heatmap illustrating the expression patterns of genes within the *Hoxa-Hoxd* clusters (left), and the associated binding of nFGFR1, RXR and Nur77 to the proximal promoter (right). **(B)** Expression of *Hoxa* genes in the presence of dominant-negative nuclear FGFR1(SP-/NLS)(TK-). mRNA expression was measured by RT-qPCR, in extracts from ESCs that had been transfected with either β-gal (control) or FGFR1(SP-/NLS)(TK-) and were then maintained in the presence of +LIF or +RA for 48 hours. In the presence of LIF, blocking nFGFR1 increased the levels of expression of the *Hoxa1*, *4* and *7* mRNAs. In RA-treated ESC, blocking nFGFR1 significantly reduced the RA-induced expression of all genes of the *Hoxa* cluster other than *Hoxa5*. * p-value <0.05; * relative to Bgal+LIF; + relative to β-gal+RA. **(C)** Expression of *Hoxa* genes in the presence of nFGFR1. nFGFR1 activates *Hoxa* gene expression to an extent similar to RA treatment. In the presence of LIF, FGFR1(SP-/NLS) induced significant upregulation of *Hoxa1* and *Hoxa2*. * p value <0.05; * relative to Bgal+LIF; **(D)** RA-induced expression of *Hoxa* cluster genes in the presence of nuclear FGFR1(SP-/NLS). In the presence of RA, transfection of FGFR1(SP-/NLS) led to a marked increase in the expression of all *Hoxa* cluster genes. P-value <0.05; * relative to β-gal+LIF; + relative to β-gal +RA.

Whereas in ESCs nFGFR1 binding was detected only at the *Hoxd1* and *Hoxd13* genes, in NCs it bound at *Hoxa1*, *2* and *4*, as determined by both ChIP-seq experiments (Fig 6A and S8A Fig) and independent ChIP assays (S8B Fig). nFGFR1 also bound to genes in downstream *Hox* clusters: *Hoxb9*, *Hoxc6*, *Hoxc8*, *Hoxd1*, and *Hoxd9* (Fig 6A and S8A Fig). RXR, in contrast, bound to the promoters in ESCs and vacated them in NCs, particularly within the *Hoxa* cluster (Fig 6A and S8A Fig), suggesting that RXR acts as a repressor and nFGFR1 as an activator of the same *Hox* genes.

We used RT-qPCR to identify the role of nFGFR1 binding in regulating *Hox* genes, analyzing the mRNA levels of the 3’ cluster (*Hoxa1-7*), which includes *Hoxa1*, critical for activating downstream *Hoxa* and *Hoxb* genes [56]. In ESCs, transfection of FGFR1(SP-/NLS)(TK-) led to increases in the levels of the *Hoxa1*, *4* and *7* mRNAs (Fig 6B), suggesting that it may repress these genes indirectly. In β-gal-transfected cells, RA-induced differentiation was accompanied by an up-regulation of the *Hoxa1-5* and *Hoxa7* mRNAs (Fig 6B), consistent with our RNA-seq experiment and earlier reports [48]. The presence of FGFR1(SP-/NLS)(TK-) abolished the RA-induced up-regulation of the *Hoxa1-5* and *Hoxa7* mRNAs, revealing a role for nFGFR1 in programming of the *Hoxa* genes (Fig 6B).

We next assessed whether nuclear accumulation of nFGFR1 is sufficient to augment the expression of *Hoxa* genes. In pluripotency medium (+LIF), FGFR1(SP-/NLS) enhanced the expression of the *Hoxa1* and *Hoxa2* mRNAs (Fig 6C). Thus, nFGFR1 can bypass LIF to activate the transcription of genes that are usually repressed in the pluripotent state. Also, FGFR1(SP-/NLS) markedly augmented the RA-induced up-regulation of all *Hoxa* mRNAs, further supporting to the notion that *Hoxa* genes are activated by nFGFR1 (Fig 6D).

CNS patterning is established by an RA gradient that is controlled by CYP26A, an RA-metabolizing protein [3, 49]. Genetic experiments have placed the *Fgfr1* gene upstream of *Cyp26*. In both our ChIP-seq experiments and independent ChIP assays (S8C and S8D Fig), nFGFR1 bound within the promoter of *Cyp26a1*, and RNA-seq as well as independent RT-qPCR assays demonstrated that it was up-regulated in NCs. Transfection of FGFR1(SP-/NLS) significantly augmented the RA-induced up-regulation of the *Cyp26a1* mRNA (S8E Fig), further indicating that the *Cyp26a1* gene is under direct control of nFGFR1.

### nFGFR1 targets and represses mesodermal genes and activates neurodevelopmental genes

An FGF/Wnt/Notch-based oscillatory mechanism is key to controlling cell differentiation and maturation in the CNS, as well positioning segmental boundaries in the developing presomitic mesoderm [3]. The *Fgfr1* gene acts upstream of both Wnt and Notch signaling to control the cyclic gene expression necessary for both developmental processes. Our data are consistent with this model, in that nFGFR1 not only targeted various neuronal genes encoding components of the Wnt/ β-catenin pathway (Fig 4F and S4D Fig), but also bound within the promoters of key mesodermal genes, including *Notch1*, *Dusp6*, *Perlecan* (H*spg2*), *Lfng*, *Nkd1*, *Nrarp*, *Mesp2* and *Porcn* (S9A Fig). As a high concentration of RA promotes neuronal but not mesodermal differentiation [28–30, 57], we tested nFGFR1 for the ability to directly repress mesodermal genes during the differentiation of ESCs into NCs. ChIP-seq experiments revealed that in NCs, nFGFR1 also bound within the *Mesp2* gene body (S9A Fig), and an independent ChIP assay confirmed this (S9B Fig). In cells transfected with control β-gal, RA treatment did not influence the expression of *Mesp2* and decreased expression of the *Notch1* and *Hspg2* mRNAs, consistent with development toward a neuronal but not mesodermal phenotype (S9C Fig). Transfection of FGFR1(SP-/NLS)(TK-) disrupted the expression of mesp2, and either antagonized or blocked the RA-induced repression of *Notch1* and *Hspg2* mRNAs (S9C Fig). In contrast, the RA-induced activation of nFGFR1 targeted neurodevelopmental genes such as *Pax3*, *Id3*, *Irx3* and *Cdx1 (* S10A Fig), was reduced by FGFR1(SP-/NLS)(TK-) (S10B Fig). Thus, endogenous nFGFR1 represses mesodermal genes and activates neural genes as ESCs progress towards a neuronal phenotype.

### nFGFR1 targets DNA motifs shared with RXR, Nur77 and other TFs

nFGFR1 lacks a DNA binding domain. However, it can associate with promoters indirectly through CBP, and thereby cooperate with various CBP-targeted TF. We used MEME-ChIP motif analysis software to identify all over-represented sequences targeted by nFGFR1, RXR or Nur77. nFGFR1-targeted motifs included the core AGGTCA sequence present in the RARE and NBRE (Table 1 and S11A Fig), as well as a consensus sequence for CREB, as we had previously shown [21, 28, 35, 42]. Related motifs for RXRα, RARa, Nurr1, RXR:Nr1h3, and PPARG (all of which contain AGGTCA) were also verified as targets for RXR and Nur77, consistent with previous reports [32–34].

**Table 1:** Over-represented DNA sequences, identified in motif analysis, reveal nFGFR1, RXR and Nur77 binding to consensus sites of diverse TFs.

Factor	MOTIF (BOLD are unique to factor)
**FGFR1**	**ARNT**, ATF1, CTCF, **ERG**/**ELK4**, **KLF4**, MAX, MZF1, NRF1, Nurr1, **Pou2f3**, **Pou5f1:Sox2**, RARα, RFX1, RXRα, **SMAD**, Sox8, SP1, STAT, **TCF3**, **TP53**, YY1, **ZBTB33**, ZFP161
**RXR**	ATF1, CTCF, **Irx4**, Mycn, MZF1, Nurr1, **Nr1h3:RXRα**, **Pitx2**, **Pou3f3**, **PPARG**, Prrx2, RARα, RFX1, RXRα, Sox8, SP1, YY1, **ZEB1**, ZFP161
**Nur77**	ATF1, CTCF, **Hic1**, MAX, Mycn, NRF1, Nurr1, **Pax6**, Prrx2, **Six6**, SP1, STAT, RXRα, YY1, ZFP161

We also detected nFGFR1 binding to TFs consensus motifs in genes encoding components of the pluripotency core [25], including KLF4, various STAT and SMAD factors, as well as motifs for multiple MYC binding partners, including ARNT, ZFP161, and MAX (Table 1 and S11A Fig). In NCs, nFGFR1 and RXR also targeted CTCF, an insulator site implicated in transcriptional activation/repression, insulation, imprinting, and inactivation of the X chromosome.

In the cases of TFs like MYC, SMAD, CTCF, KLF4, SOX2, OCT4, and STAT3, nFGFR1 interacts both with the TF-encoding genes and the consensus sequences to which they bind. This implies dual-level regulation, with nFGFR1 controlling both the generation of the TFs and its downstream function. Therefore, we next tested whether nFGFR1 influences the activities of TFs bound to additional consensus motifs, with a particular focus on a potential nFGFR1 repressor function at core pluripotency genes. Specifically, we co-transfected cells with the FGFR1 (SP-/NLS) construct, which encodes a constitutively active nFGFR1, and a luciferase reporter construct containing motifs for the binding of pluripotency TFs, including SMAD (SMAD binding element (SBE1-4)) and MYC (E-Box). Transfection of FGFR1(SP-/NLS) markedly reduced SMAD3-dependent transcriptional activity in NCs (S11B Fig), indicating that nFGFR1 indeed acts as repressor. In the case of MYC-by contrast, FGFR1(SP-/NLS) enhanced transcription in both the ESCs and NCs (S11C Fig).

nFGFR1 targeting of both the *Smad1* gene and SBE-4, as well as of the *Myc* genes and E-BOX, support the concept of dual-level transcription control by nFGFR1, i.e., (i) targeting a TF-encoding gene, and (ii) control the trans-acting function of that TFs.

## Discussion

The present study establishes nFGFR1 as a global factor that controls the genome by binding the promoters of thousands of genes, and consensus DNA sequences of diverse TF families. As such, it identifies a previously unknown, multifaceted form of control of major ontogenic pathways and gene networks.

nFGFR1 targets genes on all mouse chromosomes in a nonrandom manner, and has a distribution similar to those of two established nFGFR1 partner TFs: RXR and Nur77. Like the binding sites of RXR and Nur77, those of nFGFR1 were present throughout the genome, but most highly concentrated within regulatory regions. For example, upstream and bidirectional promoters and 5’ UTRs were frequently bound in both ESCs and NCs, and >85% of all nFGFR1 peaks were found within the proximal promoters (-1kb to +1kb TSS) of expressed genes (S2 Fig). These data corroborate our previous findings that nFGFR1 interacts with transcriptionally active chromatin in live cells, that it binds and activates the common transcription coactivator CBP, and that its binding coincides with and regulates global RNA synthesis [20, 21, 28, 58]

One feature that distinguishes the interactions of nFGFR1 with the genome from those of RXR and Nur77 is the several-fold increase in the number of promoter sites occupied by nFGFR1 only in genes that are differentially regulated in the course of RA-triggered differentiation (Fig 3B). Also, the number of genes co-targeted by nFGFR1 and either or both RXR or Nur77 increased markedly following differentiation, despite the overall loss of RXR and Nur77 binding. Thus, nFGFR1 emerges as an active mediator of RA-induced gene programing, consistent with the instructive function of nFGFR1 in the differentiation of ESCs into NCs [28], and in neuronal development *in vivo* [17].

The roles of nFGFR1, together and independently of RXR and Nur77, are further illustrated by the overlapping lists of recognition motifs in the target DNAs of these proteins (Table 1 and S11A Fig). nFGFR1 associates with sequences that are bound the classical nuclear receptors Nur77 and/or RXR, but also others that are not. nFGFR1-targeted sites encompass the consensus sequences of diverse TFs, all of which interact with CBP, and thus may engage in the nFGFR1-CBP mediated transcriptional regulation. The extensive list of nFGFR1-targeted motifs highlights how widespread use of the mechanism underlying RA-initiated gene regulation is, and the vast size of the population of responsive genes. The discovery that this regulation involves a plethora of TFs beyond RAR/RXR and Nurs reveals RA-induced transfer and retention of nFGFR1 in the nucleus [28], and nFGFR1 gene targeting to represent a global paradigm of gene programing.

Our experiments using dominant-negative and constitutively active nFGFR1 mutants have established the role of nFGFR1 as a repressor of genes in the pluripotency network during neuronal differentiation. Inactivation of the *Klf4*, *Sox2*, *Stat3*, *E2f1*, *Esrrb and Suz12*, *Smad1*, *Zfx*, *Tcfcp2l1*, and *Ctcf* genes during RA-induced differentiation appears to be mediated by the recruitment of nFGFR1 to the proximal promoter, and the disassociation of RXR and Nur77 from many of the same sites (Fig 5A and S7A Fig). These findings suggest a mechanism according to which RXR and Nur77 bind and regulate core pluripotency genes in undifferentiated cells, but nFGFR1 binds to and down-regulates the same genes during neuronal differentiation. nFGFR1 also influences the expression of all pluripotency genes examined in ESCs. However, only *Suz12*, *Myc* and *Tcfcp2l1* were directly targeted by nFGFR1 in this context.

In addition to binding promoters of the pluripotency genes, nFGFR1 interacts with the sites identified as consensus targets for the pluripotency TFs: MYC, SMAD, CTCF, KLF4, SOX2, OCT4, and STAT3. This dual-level of regulation, with nFGFR1 controlling the generation of ontogenic TFs as well as their downstream actions, may represent a feed-forward mechanism to fine tune complex ontogenic gene networks. An important nFGFR1-controlled developmental mechanism is exemplified by nFGFR1 binding both within the promoter of the *Ctcf* gene and at the CTCF consensus sequence (Fig 5C, S7A Fig and Table 1). By this dual binding, nFGFR1 may acquire control over the functions of both the *Ctcf* gene and the CTCF protein, and thus influence transcriptional insulation, pluripotency and lineage-specific gene expression due to effects on organization of higher-order chromatin structure.

Activity of the pluripotency network is opposed by the phylogenetically conserved *Hox* genes, which regulate the spatial development of differentiated organs and tissues. RA triggers the *Hox* cascade by activating transcription of the 3’ *Hoxa*-cluster genes, the most critical members of the Hox family and the first in the sequence of *Hox*-gene activation. Our results show that RA-induced nuclear accumulation of nFGFR1 and the binding of nFGFR1 to target promoters transduces the RA signal to the 3’ *Hoxa* cluster independently of RXR, a TF that vacates the promoters in this context, and also independently of Nur77, which does not bind to *Hoxa* promoters (Fig 6A, and S8A Fig). The inhibition of RA-induced *Hoxa* activation by FGFR1(SP-/NLS)(TK-) is consistent with the previously demonstrated blockade of RA-induced NC differentiation (Fig 6B) [28]. Also, overexpression of active nuclear FGFR1(SP-/NLS) is sufficient to activate the *Hox* genes, as well as neuronal development in [28] the absence of RA stimulation (Fig 6C). These findings establish nFGFR1 as a factor that programs stem-cell development, and highlight the importance of nFGFR1-mediated *Hox* gene activation. In pluripotent ESCs, the maintenance of low-level *Hoxa* activity is supported by endogenous nFGFR1. However, this support appears to be indirect, as nFGFR1 does not bind to *Hoxa*-gene promoters. It is possible that this mechanism involves SUZ12 [51], a polycomb-repressor complex that also inhibits *Hoxa* expression in ESCs prior to gastrulation [31]. Such a mechanism is supported by the fact that nFGFR1, RXR and Nur77 all target the promoter of the active *Suz12* gene in ESCs, and by the loss of RXR and Nur77 binding that accompanies *Suz12* down-regulation in differentiated NCs.

Our IPA analyses identified several additional networks of genes whose promoters are targeted by nFGFR1. These networks promote developmental processes previously known to be controlled by products of the *Fgfr1* gene, and now shown to be regulated specifically by nFGFR1. Genes whose promoters are bound by nFGFR1 in ESCs and vacated in NCs form networks that control the cell cycle and development, and that contribute to the abnormal biology of cancer cells. The postulated role of nFGFR1 in cancer [15, 23, 24] could be a consequence of nFGFR1 targeting *Tp53* and its partner genes (Fig 4B), and/or of its binding to the TP53 consensus DNA motif (Table 1 and S11A Fig). These multifaceted nFGFR1 interactions provide a new lead for cancer research, and potentially for the development of a new therapeutic target.

Genes whose promoters are targeted by nFGFR1 primarily in differentiating NCs typically regulate and execute gene expression and support embryonic, nervous-system, organ, tissue, and muscle development (Fig 4D); all of these functions are consistent with the genetically established roles of *Fgfr1*. The nFGFR1 form, in particular, has been shown to be essential and sufficient for transducing neuronal differentiation and development, and for activating neuronal genes in response to diverse receptors and second messengers [19–21, 28, 59] that converge on CREB and CBP. This function may involve binding of nFGFR1 to the promoters of genes engaged in signaling by the cAMP, PKC, BMP7, dopamine and Wnt/β-catenin proteins, all of which provide inputs to the CREB/CBP pathway. Likewise, the nFGFR1-dependent, neuronal development and regeneration induced by BMP7 or NGF [18, 28] may involve nFGFR1 targeting of the *Smad* effector genes, as well as the Semaphorin 6D, Ephrins, Ephrin receptor, and α3-integrin genes, all of whose products act in the axonal guidance pathway.

Consistent with nFGFR1-mediated neuronal specification of RA-programmed NCs [28], nFGFR1 activates neurodevelopmental genes while repressing mesodermal genes of the Wnt/ β-catenin pathway. The patterning and regional cell specification of the CNS involves dynamic regulation of *Notch1* activity, as well of the transduction of RA morphogenic signaling by *Fgfr1* [3]. The present study shows that both processes are subject to direct control by nFGFR1, which binds to promoters of the genes for *Notch1* and the RA hydrolyzing enzyme, *Cyp26a1*.

Genetic experiments have positioned FGFR1 at the top of the developmental hierarchy. The present investigation begins to answer important questions that have arisen from these findings: (1) how does disruption of a single gene, *Fgfr1*, affect multiple stages of embryonic development; (2) how does blocking nFGFR1 disrupt cellular development and the associated gene activities that are initiated by RA and other ontogenic signals; and (3) how does the constitutively active nFGFR1 protein program the differentiation of stem and progenitor cells *in vitro* and *in vivo*?

**Fig 7:**
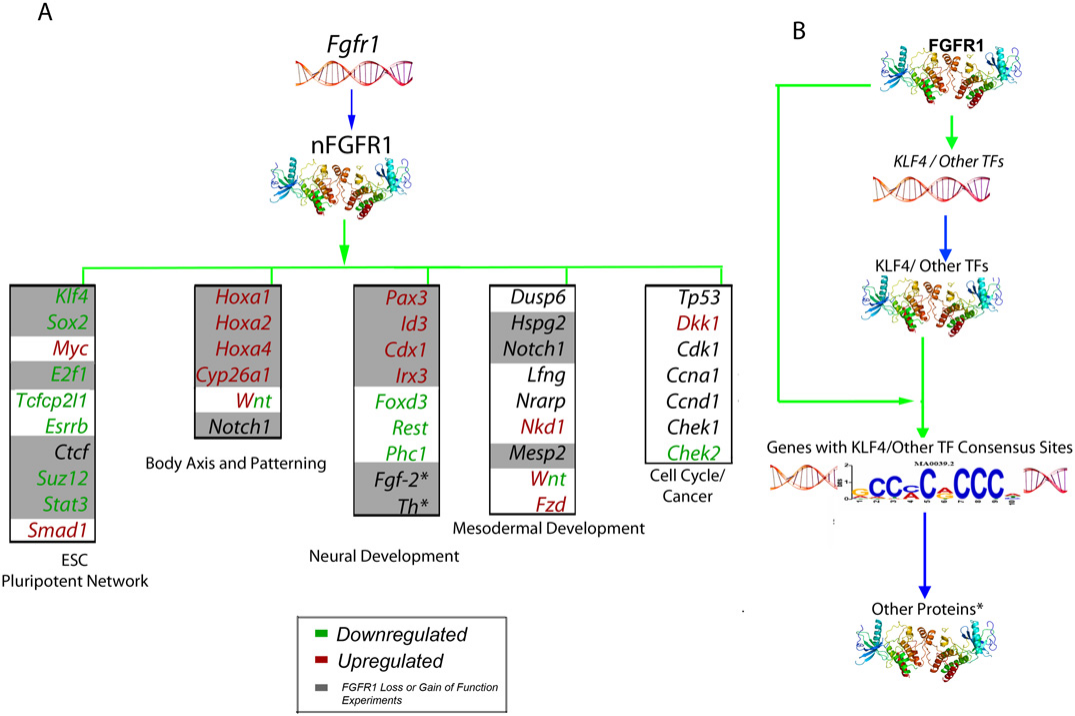
A paradigm for ontogenic global genome programming by nFGFR1. Genetic experiments position the *Fgfr1* gene at the top of gene hierarchy that directs the development of multicellular animals. *Fgfr1* governs gastrulation, as well as development of the major body axes, neural plate, central and peripheral nervous systems, and mesoderm by affecting the genes that control the cell cycle, pluripotency and differentiation [3–6], and microRNAs. This regulation is executed by a single nuclear protein, nFGFR1, which integrates signals from RA and other development-initiating factors, cooperates with RXR, Nurs and multiple TFs, and targets thousands of genes, including ones that encode miRNAs and some within top ontogenic gene networks. nFGFR1 binding to promoters of genes that encode TFs, and the genomic sequences targeted by these TFs suggest a feed forward mechanism for fine-tuning complex developmental networks. Legend: (A) nFGFR1 binding within the proximal promoter of exemplary target genes in which color denotes up- (red) or down-regulation (green) during transition from ESCs to NCs (RNA-seq). Black indicates genes that are not differentially regulated according to our cutoff. Gray box denotes direct regulation by nFGFR1, as determined by loss- or gain-of-function experiments. * denotes gene directly regulated by nFGFR1 from previous studies. (B) In the cases of KLF4 and other TFs (TP53, SMAD, CTCF, MYC, OCT4, SOX2 and STAT3), nFGFR1 interacts both with the TF-encoding genes and the consensus sequences to which they bind. This implies a feed-forward mechanism, in which nFGFR1 controls both the generation of TFs and their downstream function to fine tune ontogenic gene networks.

This study reveals the basic components of an emerging paradigm for master ontogenic networks involved in cell pluripotency, tissue and organ development, and axis specification being controlled by nFGFR1 (Fig 7A). In association with direct gene targeting, nFGFR1 binds to response (cis) elements for many of the same TFs it encodes. This dual level of regulation may allow nFGFR1 to fine tune complex ontogenic gene networks (Fig 7A and 7B).

nFGFR1 has been proposed to act as a gate-opening factor in the feed-forward-and-gate module for control of CBP. Feed-forward loops are common in biological networks, serving as pulse generators, response-delaying circuits, signal-to-noise enhancers and signal integrators. nFGFR1-controlled feed-forward-and-gate loops are positioned at several strategic nodes that may increase the efficiency and reproducibility of ontogenic pathways. The overall importance of such mechanisms in animal development is supported by the evolutionary emergence of nuclear FGFs [9] and FGFR1 [60] in early metazoans.

## Materials and Methods

### Mouse ESC culture and differentiation

Cultures of undifferentiated mouse ESCs (E14Tg2a, *American Type Culture Collection*) were propagated as previously described [28, 61]. Briefly, pluripotent ESCs were maintained in the presence of LIF (1 unit/ml), a necessary component of the pluripotent core. To induce neuronal differentiation LIF-free monolayers were treated with 1μM RA for 2 days [28–30]. ChIP-seq, RNA-seq, miRNA-seq and independent ChIP-qPCR, RT-qPCR and luciferase experiments were performed either in nondifferentiated pluripotent ESCs (+LIF), or in RA-differentiated (+RA) neuronal cells (NCs).

### Plasmids

FGFR1(SP-/NLS), in which the SP is replaced with a canonical NLS, normally provided by the FGF-2 ligand, is a constitutively active nuclear protein which binds to CBP and nFGFR1 regulated gene promoters [19, 20, 42, 54, 62, 63]. Transfected FGFR1(SP-/NLS) induces neuronal-like differentiation in ESCs [28], human NPCs [19, 20], mouse brain NPCs *in vivo* [64], and activates neuronal genes [20, 21, 28, 35]. FGFR1(SP-/NLS)(TK-), in which the tyrosine kinase domain is deleted, acts as nuclear dominant negative receptor which binds to CBP and blocks RA-induced gene activation and neuronal differentiation and prevents binding of endogenous nFGFR1 to promoters [19–21, 28, 42, 54, 62, 63]. FGFR1(TK-) acts a dominant negative receptor in both the cytoplasm and in the nucleus [42, 54]. Plasmids from Addgene—plasmid 16495: SBE4-Luc contains four copies of Smad binding element (GTCTAGAC) in pBV-Luc vector; Plasmid 11742: pCMV5B-Flag-Smad3 expression vector; Plasmid 16057: p4RTK GL4.10 (Luc2) contains three repeats of 5'-TTGGGAGGCAGCAGGTG-3' Multimerized E-box.

### Antibody verification

The nuclear presence of FGFR1 was demonstrated in several laboratories in non-transformed cells [63, 65], cancer cell lines [19, 54, 66–68], stem cells and in the rat and mouse brain [69, 70] [19, 20, 71] using an array of antibodies which target different FGFR1 epitopes. Furthermore, transfected FGFR1-EGFP in live cells was detected using native fluorescence and FGFR1-Flag using αFlag. Gene targeting by FGFR1 was shown by EMSA and ChiP assays using diverse FGFR1 antibodies: SC121, Abcam ab10646, FGFR1McAb6, Abcam FGFR1 Mab; (SC 121G) [20, 21, 24, 28, 35, 42]. FGFR1-DNA interaction was not detected in cells that do not express endogenous FGFR1 and restored by transfection of FGFR1 [20]. Gene binding by FGFR1-flag was verified with αFlag[28]. Dynamic transcription-dependent interaction of FGFR1-EGFP with chromatin was shown in live cells by confocal microscopy and FRAP[58]. In the present study, we used ChiP-validated αFGFR1, Abcam ab10646 [21, 28, 59]. Detection of nFGFR1 by this antibody is prevented by siRNA knock down of FGFR1 mRNA [23]. The ChiP validated RXR (DeltaN197, sc-774), and Nur77 (sc-5569) antibodies were purchased from Santa Cruz Biotechnology (Santa Cruz, CA) and used as previously in ChIP assays [21, 28, 35].

### ChIP assays

ESC or NC monolayers on 100mm plates were cross-linked with 1% formaldehyde (Sigma, St Louis, MO) for 10 min while shaking at room temperature and rinsed three times with cold phosphate-buffered saline. Cells from five plates were pooled and harvested in phosphate-buffered saline with protease inhibitors by 10 min centrifugation (3,000 rpm) at 4°C. ChIP was performed with 112 μg of chromatin and 3 μg of FGFR1, RXR or Nur77 antibodies using the Invitrogen MAGnify and Millipore kits, according to manufacturer’s instructions with slight modifications. Genomic DNA was precipitated with ethanol, treated with RNase A and proteinase K, and purified using the Qiagen PCR purification kit. For ChIP-seq, chromatin was further prepared using the Tru-seq ChIP Sample Preparation Kit and purified library DNA was captured on an Illumina flowcell for cluster generation and sequenced on an Illumina Hisequation 2000, following the manufacturer's protocols. In a pilot ChiP-seq experiment and in independent ChiP assays (for example S5E Fig), chromatin incubation with control IgG did not result in chromatin precipitation.

### RNA-seq, miRNA-seq and relative RNA level determination

For RNA-seq, RNA from three sets of replicate samples were processed and analyzed separately. Total RNA was isolated from two 60mm plates of ESC cultures using Trizol or the Qiagen RNA extraction kit according to manufacturer’s instructions. RNA was prepared using the Tru-Seq RNA kit and purified library cDNA was captured on an Illumina flowcell for cluster generation and sequenced on an Illumina Hisequation 2000, following the manufacturer's protocols. For independent mRNA assays, cDNA synthesis was carried out using 1 μg of RNA and the iScript cDNA Synthesis Kit (Bio-Rad; Hercules CA). One tenth of the synthesized cDNA was used as the template for real time-quantitative PCR. Twenty-five μl real time PCR reactions were performed on the BioRad MyiQ Cycler with iQ SYBR Green Supermix (Bio-Rad). RT-qPCR using the amplification cycles: Initial denaturation for 8:30 min at 95°C, followed by 35x cycle 2 (denaturation for 15 s at 95°C and annealing for 1 min at 60°C). Melt curve data collection was enabled by decreasing the set point temperature after cycle 2 by 0.58°C. The specificity of amplicons was confirmed by generating the melt curve profile of all amplified products. Gene expression was quantified as described in [72]. All oligonucleotide sequences for RT-qPCR can be found in S4 Table.

### DNA Transfections and Promoter Assays

Cells were transfected with DNA using Lipofectamine 2000 and luciferase activity was measured using the Promega Luciferase Assay System on a BioTek Plate Reader, as described previously [20, 21, 28, 42]. Luciferase experiments were repeated at least 2 times and each was performed in quadruplicate. For SBE-4 luciferase, ESCs were transfected with 250 ng of the SBE-4 construct, 250 ng of Smad3 and 250 ng of FGFR1(NLS). For E-Box luciferase, ESCs were transfected with 200 ng of the E-BOX construct and 300 ng of FGFR1(NLS). The total amount of DNA per well was adjusted to 1 μg using β-gal. For all RT-qPCR experiments, ESCs were transfected using 8 μg of either β-gal, FGFR1(SP-/NLS), FGFR1(SP-/NLS)(TK-) or FGFR1(TK-). Co-transfections of luciferase constructs with FGFR1 effector plasmids or control β-gal, had no effect on the number of transfected cells or the amount of transfected intracellular luciferase DNA measured by Real Time PCR.

### qPCR analysis of ChiP

qPCR was used to determine the relative amount of specific loci in IP, Input, and IgG (Preimmune) samples. qPCR was performed using iQ SYBR Green Supermix (Bio-Rad) on a Bio-Rad iCycler, as described above. Two and half μl of ChIP, IgG and 10% total input DNA was used in duplicate reactions of at least two independent experiments. Data are expressed as % Input. All oligonucleotide sequences for ChIP-qPCR can be found in S5 Table.

### ChIP-seq and RNA-seq data processing

For ChIP-seq, raw FASTQ reads were aligned to the mouse genome build mm10 using Bowtie 1.0 software with the following command: bowtie—m 1—p—q—S. All peaks we called were from SAM files using Model-based Analysis of ChIP-Seq (MACS) version 2.0 default settings (p-value <1e-5) with the following command: macs2 callpeak—p—t—f—g—n—B. MACS2 uses a dynamic Poisson distribution to calculate peaks.

RNA-seq was analyzed using the Tuxedo pipeline [73]. Raw FASTQ reads were mapped to the mouse mm10 genome using TopHat-2.0.8 and Bowtie2-2.1.0 software to generate SAM files. TopHat is a fast read-mapping algorithm, which uses the ultrafast Bowtie aligner for aligning reads from RNA-Seq to the reference genome. These mapped reads were assembled into transcripts and gene expression levels were calculated and normalized using Cufflinks version 2.0.2. Cufflinks produces Fragments Per Kilobase of transcript per million fragments mapped (FPKM), which is proportional to transcript abundance in each sample. Transcript assemblies were merged together using Cuffmerge and Cuffdiff was used to determine significant differences in gene expression (FPKM) between ESCs and RA-treated NCs. Heatmaps of differentially regulated genes were generated using cummeRbund in R. All raw data files for ChIP- and RNA-seq have been uploaded to GEO database under the following accession number: GSE65698.

### Conservation of FGFR1, RXR and Nur77 ChIP-seq peaks

As functional genomic regions are often evolutionarily conserved between different species, we compared the evolutionary conservation of our ChIP-seq peaks with flanking non-peak regions as an indicator of good data quality and correct data preprocessing. ChIP-seq peaks were input as BED files to the 'Conservation Plot' tool in the Galaxy/Cistrome platform (http://cistrome.org/ap/) and UCSC PhastCons conservation scores were used to detemermine the average conservation score profiles around the peak centers. For Galaxy/Cistrome, genome coordinates from all peak sets were first converted as BED files to the mm9 genome assembly using the LiftOver tool from the UCSC Table browser.

### Generation of FGFR1, RXR and Nur77 peak sets

ChIP-seq tracks were generated from MACS2 output files and uploaded to the UCSC genome browser (http://genome.ucsc.edu/) using the Galaxy web based platform (https://main.g2.bx.psu.edu/). Overlapping peak sets for FGFR1, RXR and Nur77 were generated using the concatenate, cluster and subtract tools from Galaxy. To find FGFR1+RXR+Nur77 binding sites we first identified FGFR1 peaks that directly overlapped either RXR or Nur77 by a minimum of -1 base pair (bp) and clustered these into single peak sets. Intervals from the above generated FGFR1+RXR and FGFR1+Nur77 peak sets, which further overlapped each other by a minimum of-1bp, were again clustered into the single FGFR1+RXR+Nur77 peak set. To identify genomic sites in which FGFR1 was bound with RXR, but not with Nur77, we subtracted the FGFR1+RXR+Nur77 peaks from the FGFR1+RXR peak set by a minimum of 1bp using the subtraction tool. The same was done for Nur77 by subtracting the FGFR1+RXR+Nur77 peak set from the FGFR1+Nur77 peak set. To identify the genomic sites in which FGFR1 was bound alone we further subtracted all FGFR1 peaks in either ESCs or NCs that overlapped either RXR and/or Nur77 by a minimum of 1bp in their respective sample. The same method was also used for RXR and Nur77.

To verify this method of peak generation we also used the Venn diagram tool in the Galaxy/Cistrome platform to identify overlapping peak sets. Using this method we identified a nearly identical amount of overlapping peaks as used in the above method. As all peaks were converted between the mm10 and mm9 assemblies for Galaxy/Cistrome usage, this could account for some of the peak differences between the above two methods since some peaks are lost during the LiftOver process.

### Identification of bound promoters and expressed genes

A list of UCSC known genes was obtained from the UCSC Table browser. Proximal promoters were defined as-/+ 1kb from the TSS and extracted using the Get Flanks tool on the Galaxy platform. Distal promoters were extracted the same way and defined as -5kb to -1kb TSS. The gene body was defined as all genic regions excluding the introns and exons within the defined +1kb TSS promoter. Intergenic regions were defined as all regions that did not correspond with any portion of the proximal promoter, distal promoter or gene body. The genomic distribution of each factor was generated by intersecting overlapping peaks with particular genomic regions by a minimum of 20bp. Chromosomal distribution and peak density profiles for promoters and gene body for each factor were generated using the Cis-regulatory Element Annotation System (CEAS) main program in the Galaxy/Cistrome platform.

To further relate binding to gene expression, we chose to focus on binding sites found within the proximal promoter to ensure the target promoter is associated with the designated expressed gene. We identified FGFR1, RXR and/or Nur77 peaks described above, which directly overlapped the proximal promoter by a minimum of 20bp using the join tool in Galaxy. Gene names for each promoter-binding site were obtained using the ref tool in UCSC. We next combined the total expressed and non-expressed UCSC genes from our RNA-seq data with the UCSC proximal promoter binding sites for each factor using the “Vlookup” function in Microsoft excel. We further filtered these promoter sites into non-expressed, expressed and differentially regulated genes for downstream analysis.

### Ingenuity Pathway Analysis

We imported the differentially regulated genes that met the log ratio cut-off-/+ 2.0 and p-value ≤0.05 bound by each factor into Ingenuity Pathway Analysis (IPA, Ingenuity Systems; https://www.analysis.ingenuity.com) web based software. Peaks bound to genes that were significant by RNA-seq (≥1.18 FC) but not by our cut-off (≥2.0) were also uploaded for network building but were not considered differentially regulated. All peak sets were analyzed using Core Analysis with mouse as the organism and all tissues and cell lines under a stringent filter. IPA uses a repository of biological interactions and functional annotations to build relationships (i.e. genes, proteins, drugs, and diseases) and sources major NCBI databases (EntrezGene, RefSeq, OMIM disease associations), microRNA-mRNA target databases, GWAS databases, and KEGG.

For networks, uploaded molecules of interest that interact with other molecules in the Ingenuity Knowledge Base are selected as candidates for network generation. Networks are ranked based on the number of Network Eligible molecules they contain, in which the higher the score, the lower the chance of finding the observed molecules randomly. Each network score is based on the hypergeometric distribution and calculated with the right-tailed Fisher's Exact Test. The top five networks in each peak set are ordered according to their score, with the highest network displayed first.

The significant Canonical Pathways for the dataset are displayed along the x-axis and the y-axis displays the-log of p-value calculated using the right-tailed Fisher's exact test. The significance represents the probability of molecule association from each peak set with the canonical pathway than found by random chance. Up-regulated (red), down-regulated (green), and unchanged molecules (gray) are designated in each Canonical Pathway and the top 8 pathways are shown for each peak set. The top disease and biological function p-values are also calculated using the right-tailed Fisher’s exact test and the top five in each category are listed for each peak set.

### Motif Analysis

To identify motifs, FASTA data from each of the original peak sets was extracted using the Fetch Sequences tool in the Galaxy platform and uploaded to MEME-ChIP motif software. MEME-ChIP was run on all the factors using the following parameters: meme-chip-noecho-oc. -index-name index.html-time 300-db db/JASPAR_CORE_2014_vertebrates.meme-db db/uniprobe_mouse.meme-meme-mod anr-meme-minw 6-meme-maxw 30-meme-nmotifs 5-dreme-e 0.05-centrimo-score 5-centrimo-ethresh 10 Galaxy213-_DNA_.fasta. JASPER and UNIPROBE were the chosen candidate databases. Motifs with a p-value ≤002 were selected for further analysis. For FGFR1, we focused on motifs that were present in both pluripotent and/or both RA replicates.

## Supporting Information

S1 Fig Chromosomal distribution of nFGFR1, RXR and Nur77 peaks. Figures show length of individual chromosomes as % of the entire mouse genome and % of peaks found on individual chromosomes. In both ESCs and NCs, the relative distribution of peaks for (**A**) nFGFR1, **(B)** RXR and **(C)** Nur77 was highest on chromosomes 8 and 11 and lowest on chromosomes X and Y. Average Phastcon scores for **(D)** nFGFR1, **(E)** RXR and **(F)** Nur77 in ESCs and NCs display a high level of evolutionary conservation compared to non-flanking regions. As functional regions of DNA are often conserved between species, this further demonstrates the importance of nFGFR1 as a genomic regulator, and serves as an indicator of good data quality and correct data preprocessing. **(G)** UCSC genome browser views of nFGFR1, RXR and Nur77 binding within *Fgfr1*, *Fgf-2*, *Rarα* and *Rarβ*genes. (TIF)
(TIF)

S2 Fig nFGFR1, RXR and Nur77 binding within proximal promoters of expressed genes. Nearly 90% of proximal promoter (-1kb to +1kb TSS) peaks (identified by ChIP-seq) are associated with active genes (mRNA detected by RNA-seq). (TIFF)
(TIF)

S3 Fig ChIP-seq analysis of histone variant H3.3 in RA-induced NCs. **(A)** Genomic distribution of H3.3. ChIP-seq for H3.3 was performed after 48h RA-induced NC differentiation, as described. We identified a total of 10,634 H3.3 sites that were primarily localized within the proximal promoter, gene body and intergenic regions **(B)** H3.3 peak enrichment within promoter and genic regions. H3.3 peaks were enriched 8-fold within the upstream proximal promoter (-1kb), 23-26-fold within the bidirectional promoter and 5’UTR, and 5-fold within coding exons. No such enrichment was found in the downstream promoter (+1kb), the 3’UTR or introns. **(C)** Colocalization of nFGFR1 with RXR and Nur77 coincides with H3.3 in RA-induced NCs. Within the genome, only 5% of individual nFGFR1, RXR and Nur77 binding sites overlapped with H3.3. When FGFR1 was bound with either factor separately this overlap increased to approximately 25%, and when nFGFR1 was bound with both factors combined this further increased to 63%. **(D)** H3.3 incorporates into promoters targeted by nFGFR1. Within the proximal promoter of expressed genes, H3.3 was incorporated into sites containing nFGFR1 to markedly greater degree (>81%), than sites containing only RXR, Nur77 or H3.3, but not nFGFR1 (15%). **(E)** Within the proximal promoter of differentially regulated genes, a similar preferential incorporation of H3.3 was observed within sites containing nFGFR1 greater than sites containing only RXR, Nur77 or H3.3. (TIFF)
(TIF)

S4 Fig Pathways revealed by Ingenuity Pathway Analysis (IPA) based on nFGFR1 binding to genes expressed differently in ESCs and NCs. All pathways are based on proximal promoter (-1kb to +1kb TSS) binding of designated factor to differentially expressed genes (FC ≥-/+2.0 and p-value <0.05). For canonical pathways displayed in **(B)**, **(C)** and **(D)**; a pink border represents genes or groups of genes bound by nFGFR1, in which the degree of gene upregulation (red) and downregulation (green) is denoted by the color intensity. A rainbow color represents a group of genes that contains members that are both up- and down-regulated. Grey symbols represent genes bound by FGFR1 but were not differentially regulated according to our cut-off. A double border denotes a group or complex of functionally related genes within the pathway. A complete interpretation of network shapes and interactions can be found in Material and Methods. All p-values were calculated using the right-tailed Fisher’s exact test. **(A)** Genome browser views of nFGFR1, RXR and Nur77 binding within the proximal promoter of *Tp53*, *Chek2*, *Dkk1* and *Camk2d* genes in ESCs and NCs. **(B)** “CREB signaling in neurons” pathway based on nFGFR1 promoter binding in pluripotent ESCs. The top differentially regulated genes include *CamkII*, *Adenylate cyclase (AC)*, *Phospholipase C (PLC)*, and *G-protein β(Gβ)*. **(C)** “Axonal guidance signaling canonical pathway” based on nFGFR1 promoter binding in NCs. nFGFR1 targets promoters of the ephrins, ephrin receptors, integrins and BMP7 genes. **(D)** “Wnt/B-catenin pathway” based on nFGFR1 promoter binding in NC. The top differentially regulated genes include *Rar*, *Sox*, *c-Jun*, *Wnt*, D*kk1* and *Frizzled*. (TIFF)
(TIF)

S5 ig Pathways revealed by Ingenuity Pathway Analysis (IPA) based on nFGFR1, RXR and Nur77 binding to genes expressed differently in ESCs and NCs. All pathways are based on proximal promoter (-1kb to +1kb TSS) binding of designated factor to differentially expressed genes (FC ≥-/+2.0 and p-value <0.05). For canonical pathways displayed in **(D)** and **(F)**; a pink border represents genes or groups of genes bound by nFGFR1, in which the degree of gene upregulation (red) and downregulation (green) is denoted by the color intensity. A rainbow color represents a group of genes that contains members that are both up- and down-regulated. Grey symbols represent genes bound by FGFR1 but were not differentially regulated according to our cut-off. A double border denotes a group or complex of functionally related genes within the pathway. A complete interpretation of network shapes and interactions can be found in Material and Methods. All p-values were calculated using the right-tailed Fisher’s exact test. **(A)** List of top canonical pathways based on differentially regulated genes bound by FGFR1 and RXR in pluripotent ESCs. The top 8 significant pathways are shown. **(B)** Top canonical pathways are based on differentially regulated genes bound by FGFR1 and Nu77 in ESCs. The top 8 significant pathways are shown. **(C)** Top canonical pathways based on differentially regulated genes bound by nFGFR1 and RXR in NC. The top 8 significant pathways are shown. **(D)** “Role of Nanog in mammalian ESC pluripotency” pathway based on nFGFR1 and RXR binding in NCs. The top differentially regulated genes bound by nFGFR1-RXR include *Wnt*, *Frizzled*, *Bmp*, and *Smads*. **(E)** Top canonical pathways based on differentially regulated genes bound by nFGFR1 and Nur77 in NCs. The top 8 significant pathways are shown. **(F)** “Dopamine DARPP32 Feedback in cAMP signaling” pathway based on nFGFR1 and Nur77 binding in RA-induced NCs. The top differentially regulated genes include *Dopamine receptors 2/3/4*, *Protein kinase C* (PKC), *Phospholipase C (PLC)*, and *Potassium inwardly-rectifying channel* (*KCNJ*). **(G)** Top canonical pathways based on differentially regulated genes bound by nFGFR1, RXR and Nur77 in ESCs. The top 8 significant pathways are shown. **(H)** Top canonical pathways based on differentially regulated genes bound by FGFR1, RXR and Nur77 in RA-induced NCs. The top 8 significant pathways are shown. (TIFF)
(TIF)

S6 Fig ChIP-seq analysis of nFGFR1 binding in NCs. **(A)** Average Phastcon scores for nFGFR1 from second ChIP-seq replicate after 48h RA-induced NC differentiation. **(B)** Genomic distribution of nFGFR1 within the proximal promoter, distal promoter, genic and intergenic regions. We identified a total of 14,082 genomic peaks of which 11,223 were localized within the proximal promoter. **(C)** nFGFR1 peak enrichment within promoter and genic regions. nFGFR1 peaks were enriched over 30-fold in the upstream proximal promoter (-1kb), over 116- fold within the bidirectional promoter and 94-fold within the 5’UTR. No enrichment was observed in the downstream promoter, 3’UTR or introns. (TIFF)
(TIF)

S7 Fig nFGFR1 targeting and regulation of core pluripotent genes. **(A)** UCSC genome browser views of nFGFR1, RXR and Nur77 binding to promoters of core pluripotent genes (*Suz12*, *Essrb*, *Smad1*, *Klf4*, *Ctcf*, *Sox2*, *Stat3*, *E2f1*, *Tfcp2l1*, and *Zfx*) in pluripotent ESCs and RA-differentiated NCs. **(B)** Independent ChIP assay demonstrating nFGFR1 binding within the proximal promoter of the *Suz12* gene **(C)** Dominant negative FGFR1(TK-) disrupts the expression of core pluripotent genes in the presence of LIF. mRNA expression levels were measured using RT-qPCR with extracts from ESCs transfected with either β-gal (control) or FGFR1(TK-) and subsequently maintained in the presence of +LIF or +RA for 48 hours. In the presence of LIF, blocking both membrane bound and nFGFR1 increased the levels of all genes examined. However, its effect on the RA-induced downregulation of nearly all genes was markedly diminished as compared to FGFR1 (SP-/NLS)(TK-). P value <0.05 * different from β-gal+LIF; + different from β-gal+RA. (TIFF)
(TIF)

S8 Fig nFGFR1 targeting and regulation of the *Hoxa* cluster genes and the *Cyp26a1* gene. **(A)** UCSC genome browser views of nFGFR1, RXR, and Nur77 binding within the *Hoxa-Hoxd* gene cluster. **(B)** Independent ChIP assays demonstrating nFGFR1 binding within the proximal promoter of the *hoxa1*, *hoxa2* and *hoxa3* genes. **(C)** UCSC genome browser views of nFGFR1, RXR and Nur77 binding within the proximal promoter of *Cyp26a1*. **(D)** Independent ChIP assays demonstrating nFGFR1 binding within the proximal promoter of the *Cyp26a1* gene. **(E)** FGFR1(SP-/NLS) augments the RA-induced expression of *Cyp26a1*. mRNA expression levels were measured using RT-qPCR with extracts from ESCs transfected with either β-gal (control) or FGFR1(SP-/NLS) and subsequently maintained in the presence of +LIF or +RA for 48 hours. In the presence of RA, transfection of FGFR1(SP-/NLS) significantly augmented the expression of *Cyp26a1*. P value <0.05 * different from β-gal+LIF; + different from β-gal+RA. (TIFF)
(TIF)

S9 Fig nFGFR1 binding and regulation of key mesodermal genes. **(A)** UCSC Genome browser views of nFGFR1, RXR, and Nur77 binding to within the promoter of *Notch1*, *Perlecan* (*Hspg2*), Dusp6, *Lfng*, *Nkd1*, *Nrarp*, *Pornc and Mesp2*. **(B)** Independent ChIP assay showing nFGFR1 binding within the *Mesp2* gene body (**C)** FGFR1(SP-/NLS)(TK-) disrupts the expression of Mesp2 and antagonizes or blocks the RA-induced repression of *Notch1* and *Hspg2* mRNA expression. mRNA expression levels were measured using extracts from ESCs transfected with either β-gal (control) or FGFR1(SP-/NLS)(TK) and subsequently maintained in the presence of +LIF or +RA for 48 hours. P value <0.05 * different from β-gal+LIF; + different from β-gal+RA. (TIFF)
(TIF)

S10 Fig nFGFR1 binding and regulation of key neuronal genes. **(A)** UCSC genome browser views of nFGFR1, RXR and Nur77 binding within the promoter of *Pax3*, *Id3*, *Cdx1* and *Irx3*. **(B)** FGFR1(SP-/NLS) (TK-) antogonizes or blocks the RA-induced activation of *Pax3*, *Id3*, *Cdx1* and *Irx3*. mRNA expression levels were mesured using extracts from ESCs transfected with either β-gal (control) or FGFR1(SP-/NLS) (TK-) and subsequently maintained in the presence of +LIF or +RA for 48 hours. P value <0.05 * different from β-gal+LIF; + different from β-gal+RA. (TIFF)
(TIF)

S11 Fig Over-represented DNA sequences targeted by nFGFR1, RXR and Nur77. **(A)** All motif analyses were carried out using MEME-ChIP software. The over-represented DNA sequences reveal nFGFR1 binding to over 30 motifs representing consensus sequences of diverse TFs, all of which are known to interact with nFGFR1 binding partner and transcriptional coactivator CBP. Consistent with partial overlap of nFGFR1 peaks with RXR and/or Nur77, we found a number of the consensus sequences in which (i) nFGFR1 shares with RXR and/or Nur77 (ATF1,CTCF, MAX, NZF1, RXRα/Nur1, NRF1, RARα, RFX1, SMAD3, SOX8, SP1, STAT1, STAT3, YY1, ZFP161), (ii) consensus sequences targeted by nFGFR1 alone (ARNT, ERG/ELK4, KLF4, POU2F3, POU5F1/SOX2, SMAD2/4, TCF3, TP53, ZBTB33) and (iii) consensus sequences targeted by RXR and/or Nur77 but not shared with nFGFR1 (HIC1, IRX4, Mycn, PAX6,PITX2, POUF3, PPARG, PRRRX2, SIX6, ZEB1). In cases of TFs like CTCF, Klf4, SOX2, STAT3, and TP-53, nFGFR1 interacts both with their cognate DNA sequences as well as genes that encode these TFs. This implies a dual-level of regulation in which nFGFR1 controls generation of TFs as well as their downstream functions. **(B)** FGFR1(SP-/NLS) inhibits SBE-4 activation in the presence of Smad3. mESCs were transfected with the SBE-4-luc construct and either β-gal, FGFR1 (SP-/NLS), SMAD3 or SMAD3+FGFR1(SP-/NLS), and subsequently maintained in the presence of +LIF or +RA for 24 hours. Luciferase activity was normalized to β-gal. In the presence of RA, nuclear active FGFR1(SP-/NLS) significantly decreased the SMAD3-dependent activation of SBE4-luc. **(C)** FGFR1 (SP-/NLS) augments E-BOX activation in the presence of LIF and RA. mESCs were transfected with the E-BOX-luc construct and either β-gal or FGFR1(SP-/NLS), and subsequently maintained in the presence of +LIF or +RA for 24 hours. Luciferase activity was normalized to β-gal. Transfection of FGFR1 (SP-/NLS) significantly enhanced E-BOX dependent transcriptional activity in the presence of either LIF of RA treatment. (TIFF)
(TIF)

S1 Table nFGFR1, RXR and Nur77 peaks on mouse genome in pluripotent ESCs and differentiated NCs. (XLSX)

S2 Table RNA-seq analysis of all mRNA genes expressed in ESCs and NCs. (XLSX)

S3 Table miRNA-seq analysis and miRNA genes targeted by nFGFR1 in ESCs and NCs. (XLSX)

S4 Table Primers used in RT-qPCR assays. (DOCX)

S5 Table Primers used in ChIP-qPCR assays. (DOCX)
