# Supplementary figures and images for: Global Developmental Gene Programing Involves a Nuclear Form of Fibroblast Growth Factor Receptor-1 (FGFR1)
Source: PLoS One. 2015 Apr 29;10(4):e0123380. doi: 10.1371/journal.pone.0123380 (PMC4414453; doi:10.1371/journal.pone.0123380)

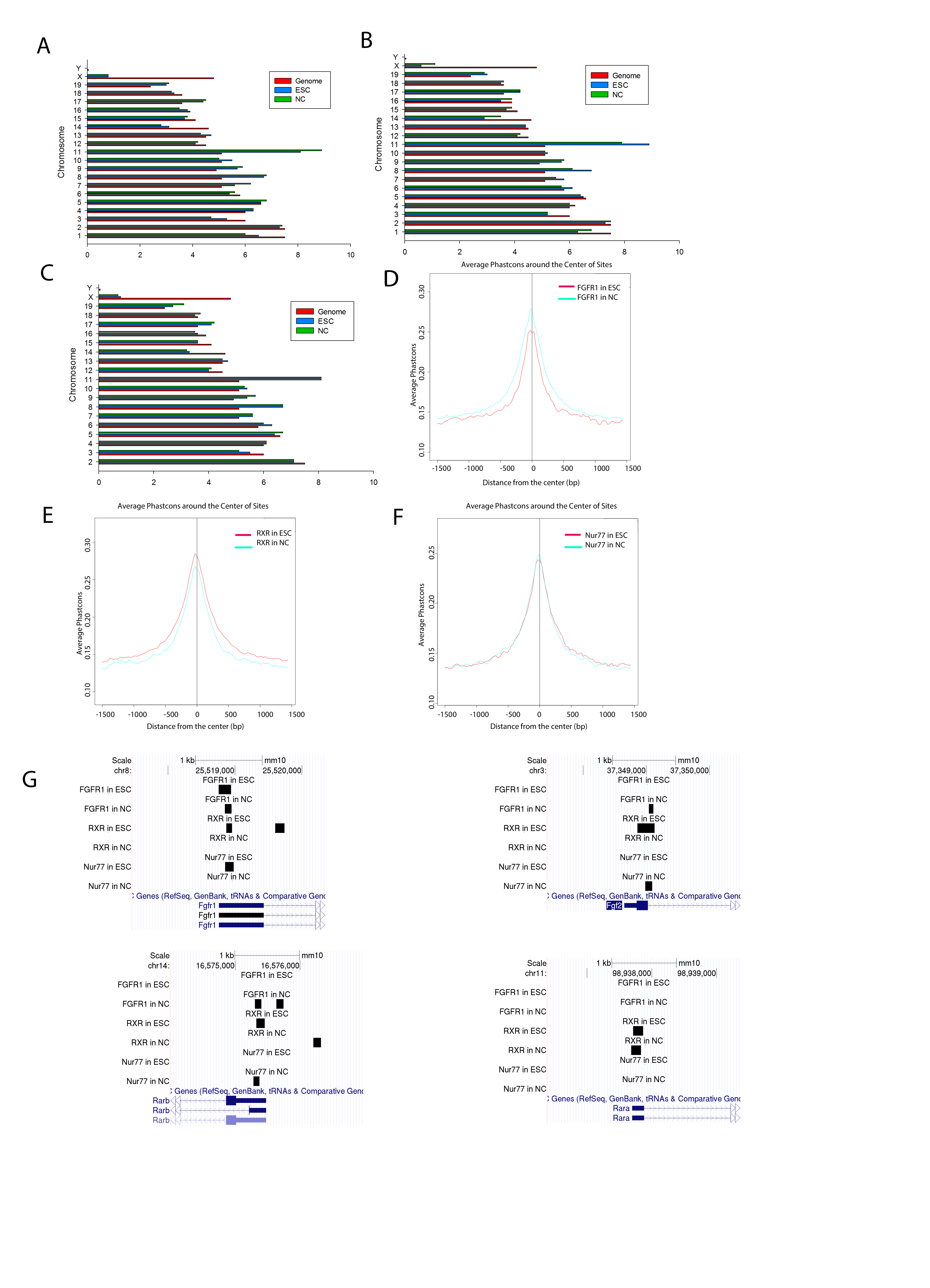

Supplement: S1 Fig — Figures show length of individual chromosomes as % of the entire mouse genome and % of peaks found on individual chromosomes. In both ESCs and NCs, the relative distribution of peaks for (A) nFGFR1, (B) RXR and (C) Nur77 was highest on chromosomes 8 and 11 and lowest on chromosomes X and Y. Average Phastcon scores for (D) nFGFR1, (E) RXR and (F) Nur77 in ESCs and NCs display a high level of evolutionary conservation compared to non-flanking regions. As functional regions of DNA are often conserved between species, this further demonstrates the importance of nFGFR1 as a genomic regulator, and serves as an indicator of good data quality and correct data preprocessing. (G) UCSC genome browser views of nFGFR1, RXR and Nur77 binding within Fgfr1, Fgf-2, Rarα and Rarβgenes. (TIF) (TIF) [file pone.0123380.s001.tif]

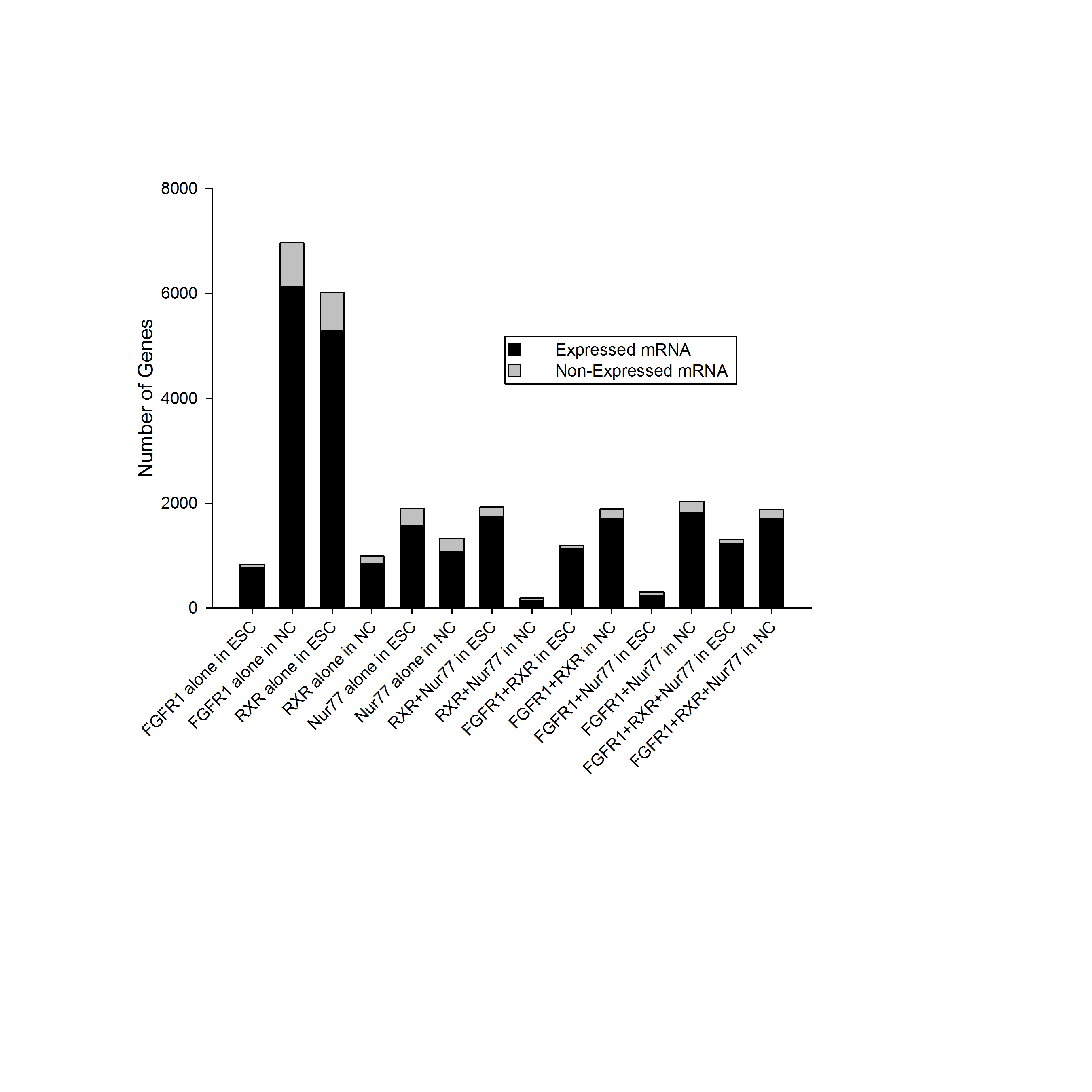

Supplement: S2 Fig — Nearly 90% of proximal promoter (-1kb to +1kb TSS) peaks (identified by ChIP-seq) are associated with active genes (mRNA detected by RNA-seq). (TIFF) (TIF) [file pone.0123380.s002.tif]

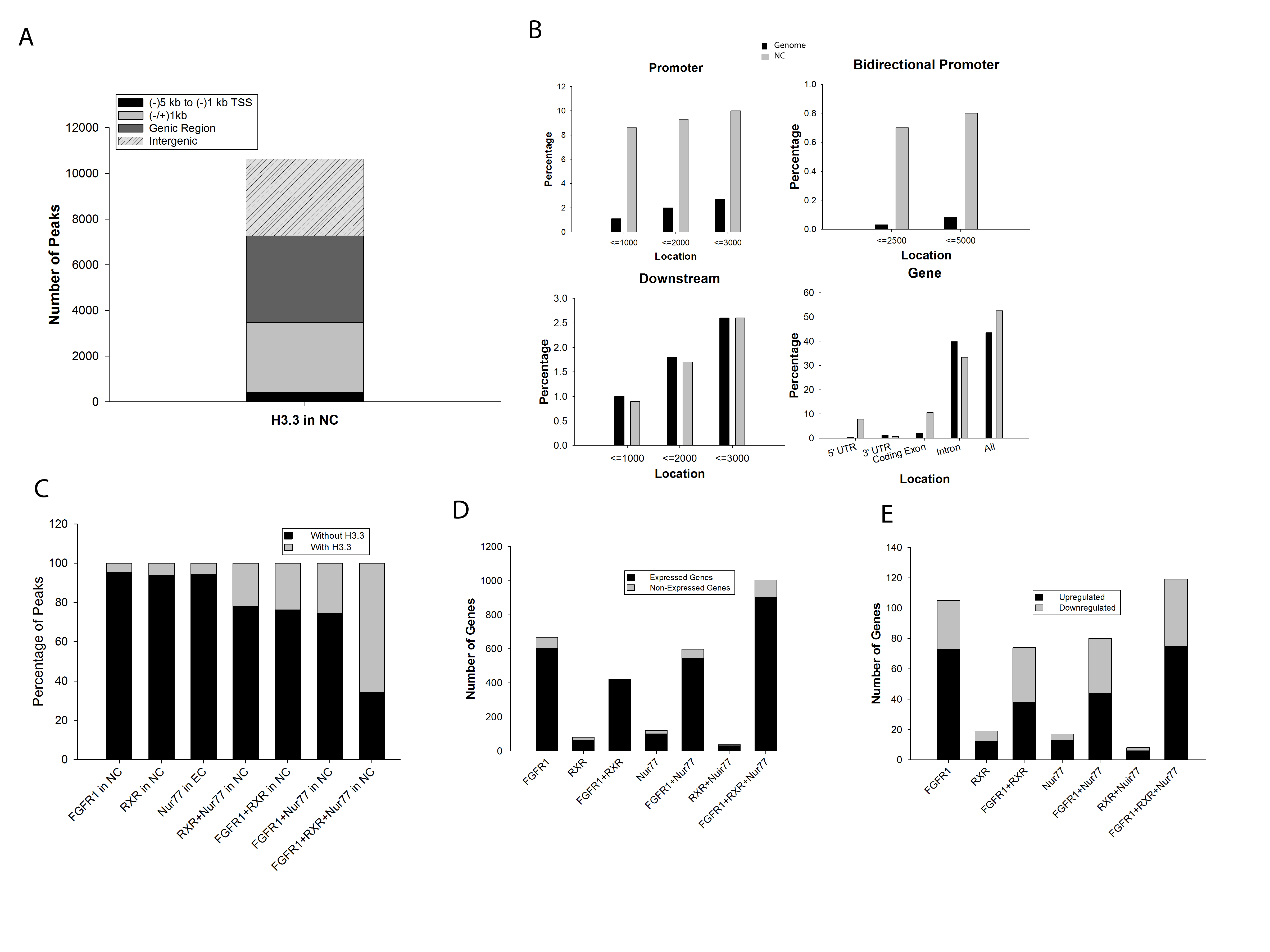

Supplement: S3 Fig — (A) Genomic distribution of H3.3. ChIP-seq for H3.3 was performed after 48h RA-induced NC differentiation, as described. We identified a total of 10,634 H3.3 sites that were primarily localized within the proximal promoter, gene body and intergenic regions (B) H3.3 peak enrichment within promoter and genic regions. H3.3 peaks were enriched 8-fold within the upstream proximal promoter (-1kb), 23-26-fold within the bidirectional promoter and 5’UTR, and 5-fold within coding exons. No such enrichment was found in the downstream promoter (+1kb), the 3’UTR or introns. (C) Colocalization of nFGFR1 with RXR and Nur77 coincides with H3.3 in RA-induced NCs. Within the genome, only 5% of individual nFGFR1, RXR and Nur77 binding sites overlapped with H3.3. When FGFR1 was bound with either factor separately this overlap increased to approximately 25%, and when nFGFR1 was bound with both factors combined this further increased to 63%. (D) H3.3 incorporates into promoters targeted by nFGFR1. Within the proximal promoter of expressed genes, H3.3 was incorporated into sites containing nFGFR1 to markedly greater degree (>81%), than sites containing only RXR, Nur77 or H3.3, but not nFGFR1 (15%). (E) Within the proximal promoter of differentially regulated genes, a similar preferential incorporation of H3.3 was observed within sites containing nFGFR1 greater than sites containing only RXR, Nur77 or H3.3. (TIFF) (TIF) [file pone.0123380.s003.tif]

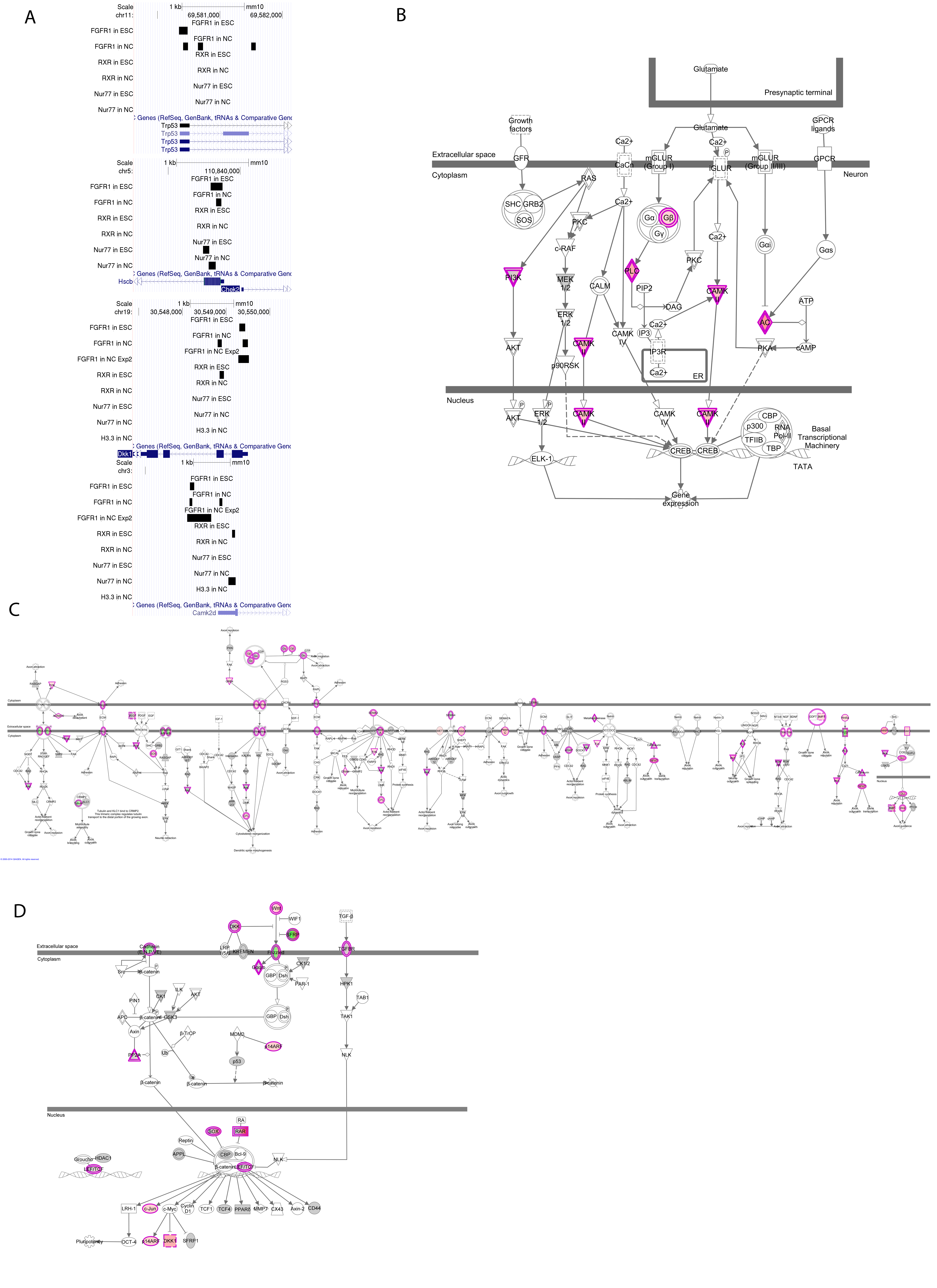

Supplement: S4 Fig — All pathways are based on proximal promoter (-1kb to +1kb TSS) binding of designated factor to differentially expressed genes (FC ≥-/+2.0 and p-value <0.05). For canonical pathways displayed in (B), (C) and (D); a pink border represents genes or groups of genes bound by nFGFR1, in which the degree of gene upregulation (red) and downregulation (green) is denoted by the color intensity. A rainbow color represents a group of genes that contains members that are both up- and down-regulated. Grey symbols represent genes bound by FGFR1 but were not differentially regulated according to our cut-off. A double border denotes a group or complex of functionally related genes within the pathway. A complete interpretation of network shapes and interactions can be found in Material and Methods. All p-values were calculated using the right-tailed Fisher’s exact test. (A) Genome browser views of nFGFR1, RXR and Nur77 binding within the proximal promoter of Tp53, Chek2, Dkk1 and Camk2d genes in ESCs and NCs. (B) “CREB signaling in neurons” pathway based on nFGFR1 promoter binding in pluripotent ESCs. The top differentially regulated genes include CamkII, Adenylate cyclase (AC), Phospholipase C (PLC), and G-protein β(Gβ). (C) “Axonal guidance signaling canonical pathway” based on nFGFR1 promoter binding in NCs. nFGFR1 targets promoters of the ephrins, ephrin receptors, integrins and BMP7 genes. (D) “Wnt/B-catenin pathway” based on nFGFR1 promoter binding in NC. The top differentially regulated genes include Rar, Sox, c-Jun, Wnt, Dkk1 and Frizzled. (TIFF) (TIF) [file pone.0123380.s004.tif]

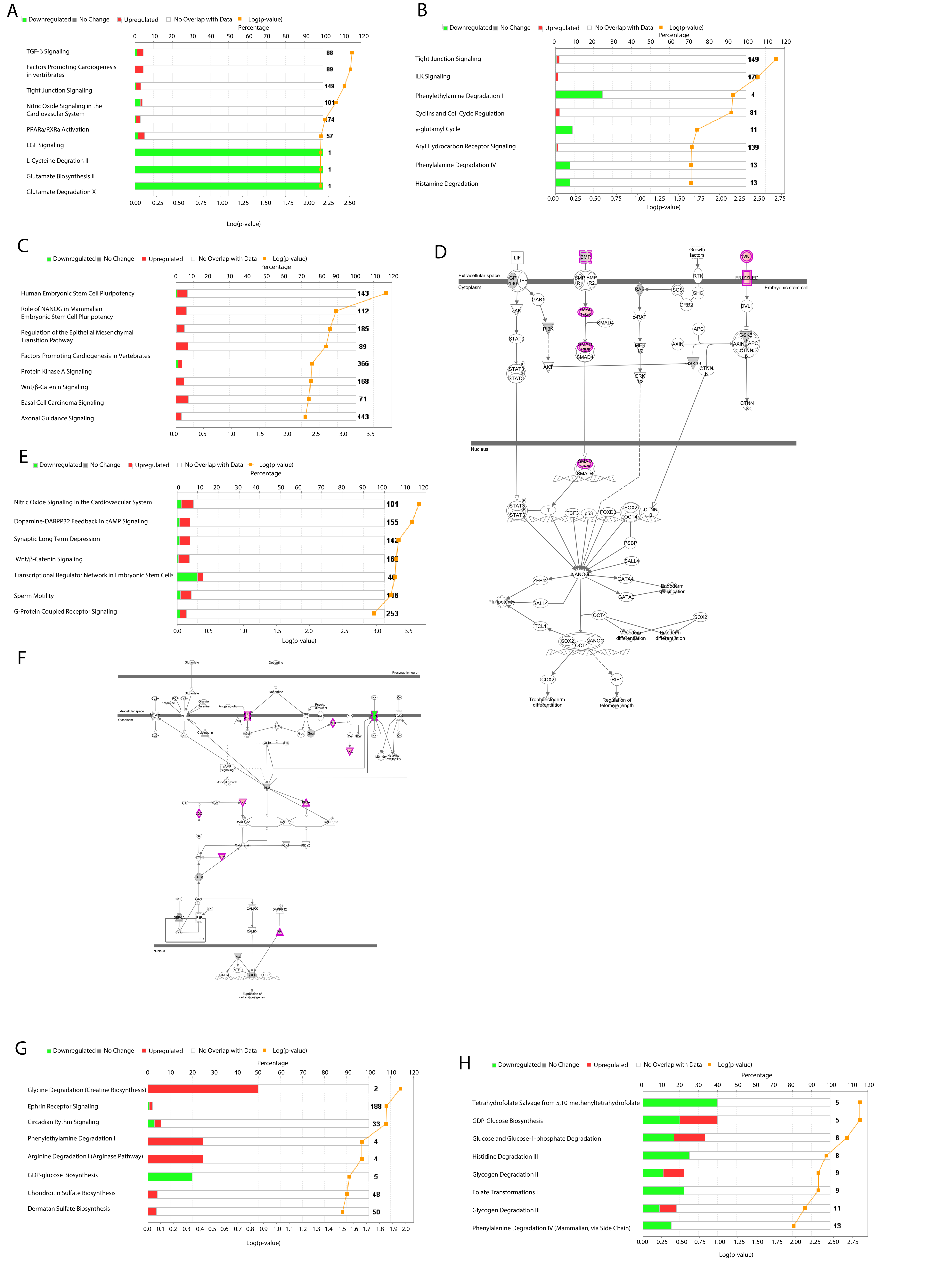

Supplement: S5 ig — All pathways are based on proximal promoter (-1kb to +1kb TSS) binding of designated factor to differentially expressed genes (FC ≥-/+2.0 and p-value <0.05). For canonical pathways displayed in (D) and (F); a pink border represents genes or groups of genes bound by nFGFR1, in which the degree of gene upregulation (red) and downregulation (green) is denoted by the color intensity. A rainbow color represents a group of genes that contains members that are both up- and down-regulated. Grey symbols represent genes bound by FGFR1 but were not differentially regulated according to our cut-off. A double border denotes a group or complex of functionally related genes within the pathway. A complete interpretation of network shapes and interactions can be found in Material and Methods. All p-values were calculated using the right-tailed Fisher’s exact test. (A) List of top canonical pathways based on differentially regulated genes bound by FGFR1 and RXR in pluripotent ESCs. The top 8 significant pathways are shown. (B) Top canonical pathways are based on differentially regulated genes bound by FGFR1 and Nu77 in ESCs. The top 8 significant pathways are shown. (C) Top canonical pathways based on differentially regulated genes bound by nFGFR1 and RXR in NC. The top 8 significant pathways are shown. (D) “Role of Nanog in mammalian ESC pluripotency” pathway based on nFGFR1 and RXR binding in NCs. The top differentially regulated genes bound by nFGFR1-RXR include Wnt, Frizzled, Bmp, and Smads. (E) Top canonical pathways based on differentially regulated genes bound by nFGFR1 and Nur77 in NCs. The top 8 significant pathways are shown. (F) “Dopamine DARPP32 Feedback in cAMP signaling” pathway based on nFGFR1 and Nur77 binding in RA-induced NCs. The top differentially regulated genes include Dopamine receptors 2/3/4, Protein kinase C (PKC), Phospholipase C (PLC), and Potassium inwardly-rectifying channel (KCNJ). (G) Top canonical pathways based on differentially regulated genes bound [file pone.0123380.s005.tif]

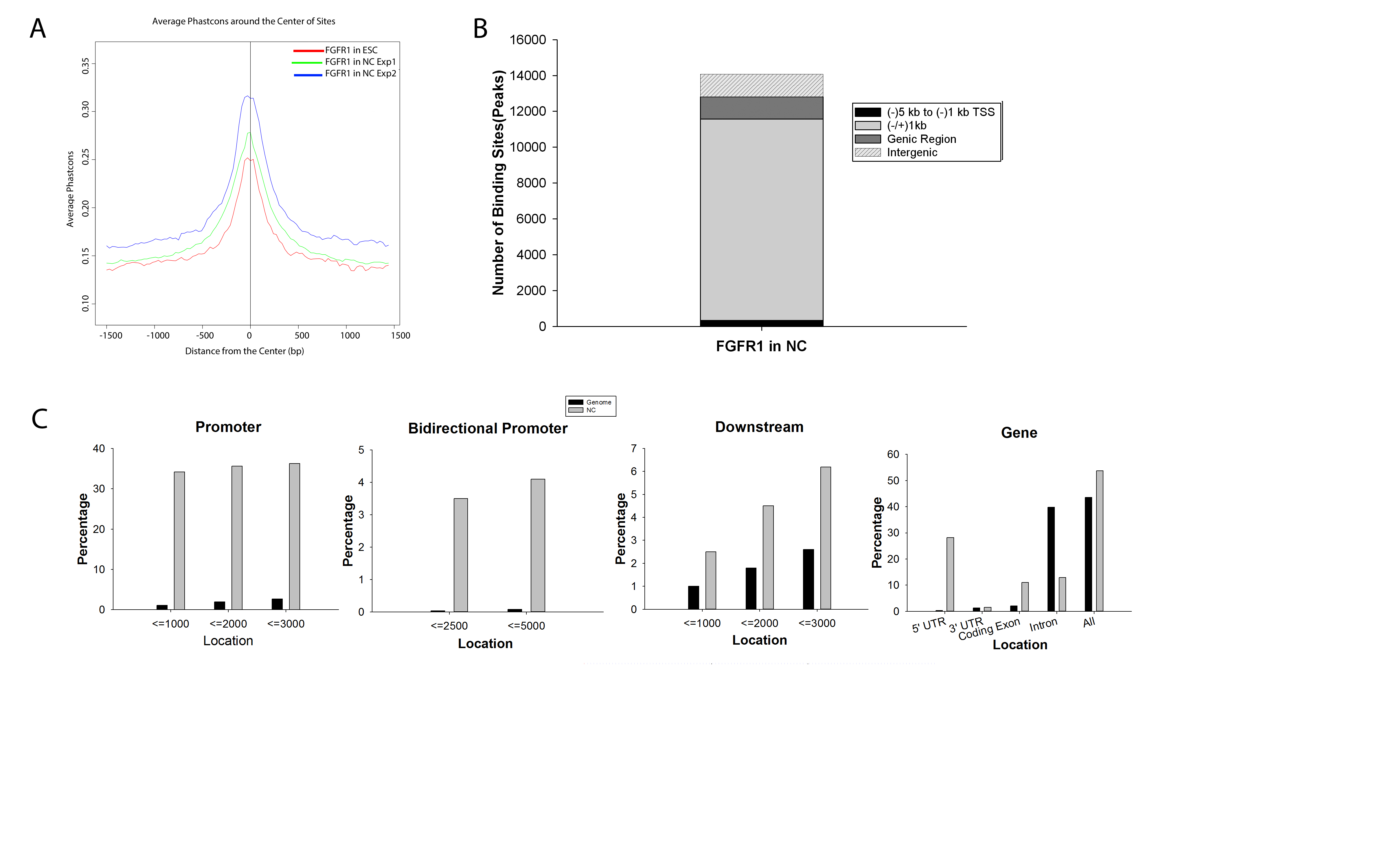

Supplement: S6 Fig — (A) Average Phastcon scores for nFGFR1 from second ChIP-seq replicate after 48h RA-induced NC differentiation. (B) Genomic distribution of nFGFR1 within the proximal promoter, distal promoter, genic and intergenic regions. We identified a total of 14,082 genomic peaks of which 11,223 were localized within the proximal promoter. (C) nFGFR1 peak enrichment within promoter and genic regions. nFGFR1 peaks were enriched over 30-fold in the upstream proximal promoter (-1kb), over 116- fold within the bidirectional promoter and 94-fold within the 5’UTR. No enrichment was observed in the downstream promoter, 3’UTR or introns. (TIFF) (TIF) [file pone.0123380.s006.tif]

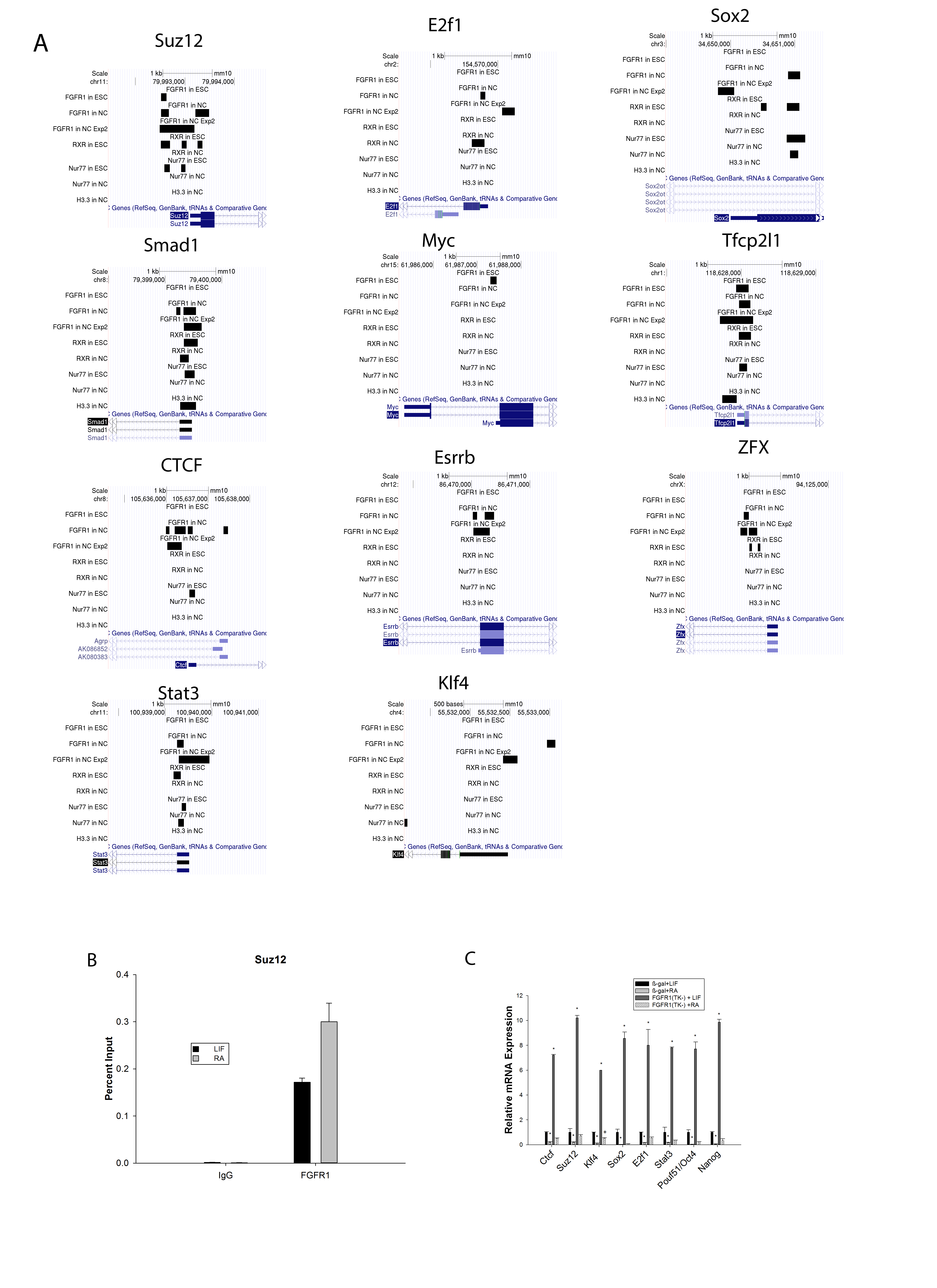

Supplement: S7 Fig — (A) UCSC genome browser views of nFGFR1, RXR and Nur77 binding to promoters of core pluripotent genes (Suz12, Essrb, Smad1, Klf4, Ctcf, Sox2, Stat3, E2f1, Tfcp2l1, and Zfx) in pluripotent ESCs and RA-differentiated NCs. (B) Independent ChIP assay demonstrating nFGFR1 binding within the proximal promoter of the Suz12 gene (C) Dominant negative FGFR1(TK-) disrupts the expression of core pluripotent genes in the presence of LIF. mRNA expression levels were measured using RT-qPCR with extracts from ESCs transfected with either β-gal (control) or FGFR1(TK-) and subsequently maintained in the presence of +LIF or +RA for 48 hours. In the presence of LIF, blocking both membrane bound and nFGFR1 increased the levels of all genes examined. However, its effect on the RA-induced downregulation of nearly all genes was markedly diminished as compared to FGFR1 (SP-/NLS)(TK-). P value <0.05 * different from β-gal+LIF; + different from β-gal+RA. (TIFF) (TIF) [file pone.0123380.s007.tif]

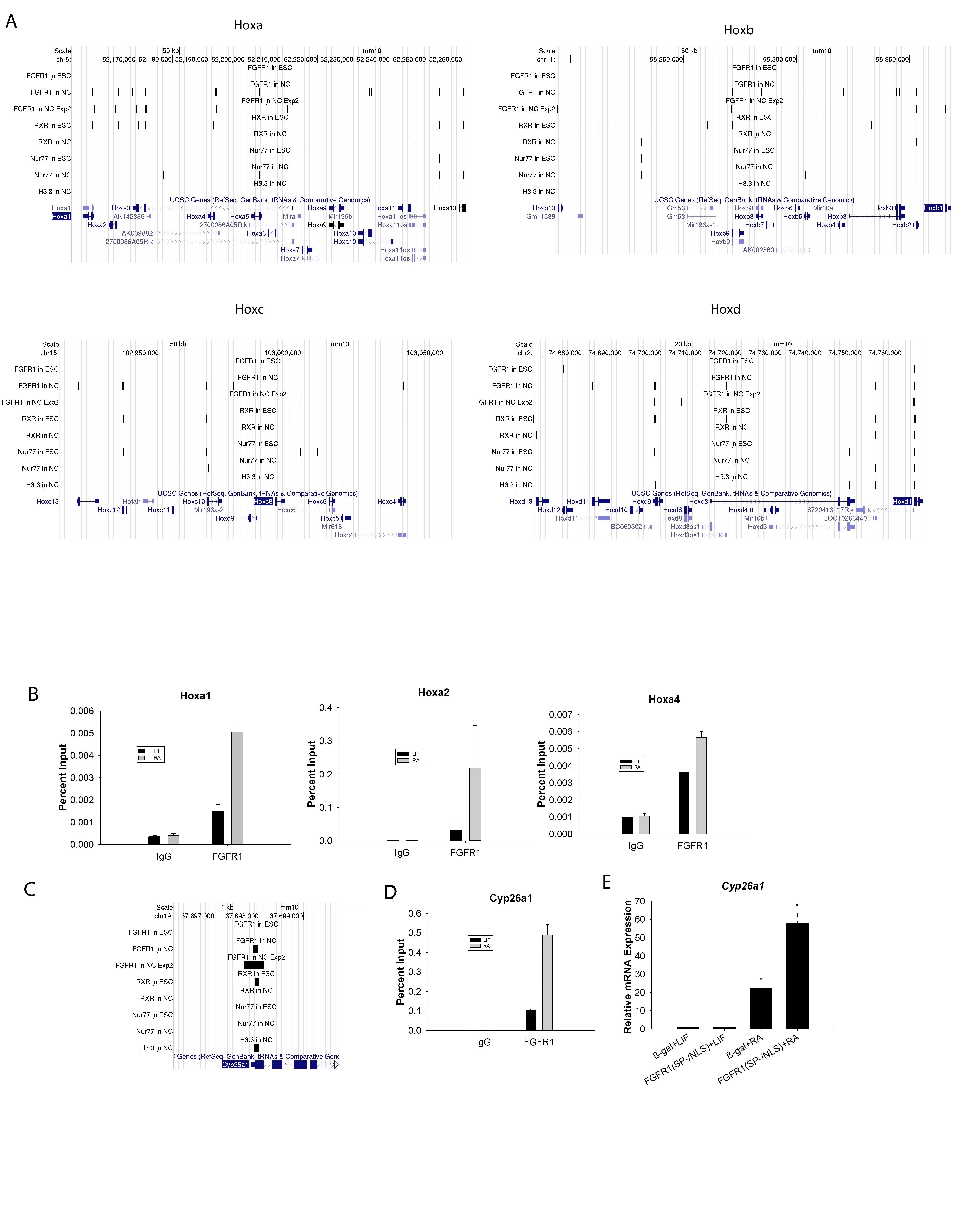

Supplement: S8 Fig — (A) UCSC genome browser views of nFGFR1, RXR, and Nur77 binding within the Hoxa-Hoxd gene cluster. (B) Independent ChIP assays demonstrating nFGFR1 binding within the proximal promoter of the hoxa1, hoxa2 and hoxa3 genes. (C) UCSC genome browser views of nFGFR1, RXR and Nur77 binding within the proximal promoter of Cyp26a1. (D) Independent ChIP assays demonstrating nFGFR1 binding within the proximal promoter of the Cyp26a1 gene. (E) FGFR1(SP-/NLS) augments the RA-induced expression of Cyp26a1. mRNA expression levels were measured using RT-qPCR with extracts from ESCs transfected with either β-gal (control) or FGFR1(SP-/NLS) and subsequently maintained in the presence of +LIF or +RA for 48 hours. In the presence of RA, transfection of FGFR1(SP-/NLS) significantly augmented the expression of Cyp26a1. P value <0.05 * different from β-gal+LIF; + different from β-gal+RA. (TIFF) (TIF) [file pone.0123380.s008.tif]

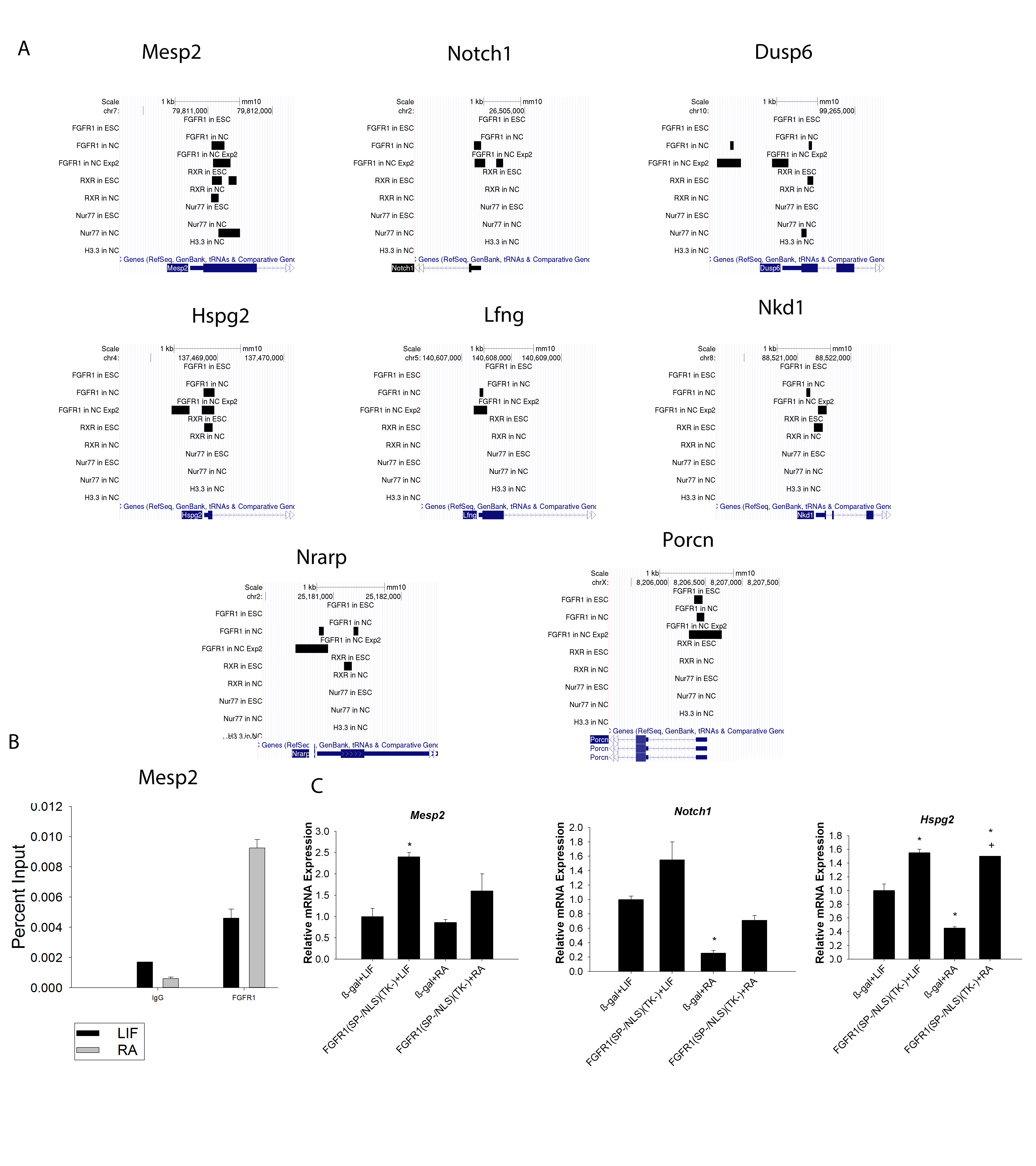

Supplement: S9 Fig — (A) UCSC Genome browser views of nFGFR1, RXR, and Nur77 binding to within the promoter of Notch1, Perlecan (Hspg2), Dusp6, Lfng, Nkd1, Nrarp, Pornc and Mesp2. (B) Independent ChIP assay showing nFGFR1 binding within the Mesp2 gene body (C) FGFR1(SP-/NLS)(TK-) disrupts the expression of Mesp2 and antagonizes or blocks the RA-induced repression of Notch1 and Hspg2 mRNA expression. mRNA expression levels were measured using extracts from ESCs transfected with either β-gal (control) or FGFR1(SP-/NLS)(TK) and subsequently maintained in the presence of +LIF or +RA for 48 hours. P value <0.05 * different from β-gal+LIF; + different from β-gal+RA. (TIFF) (TIF) [file pone.0123380.s009.tif]

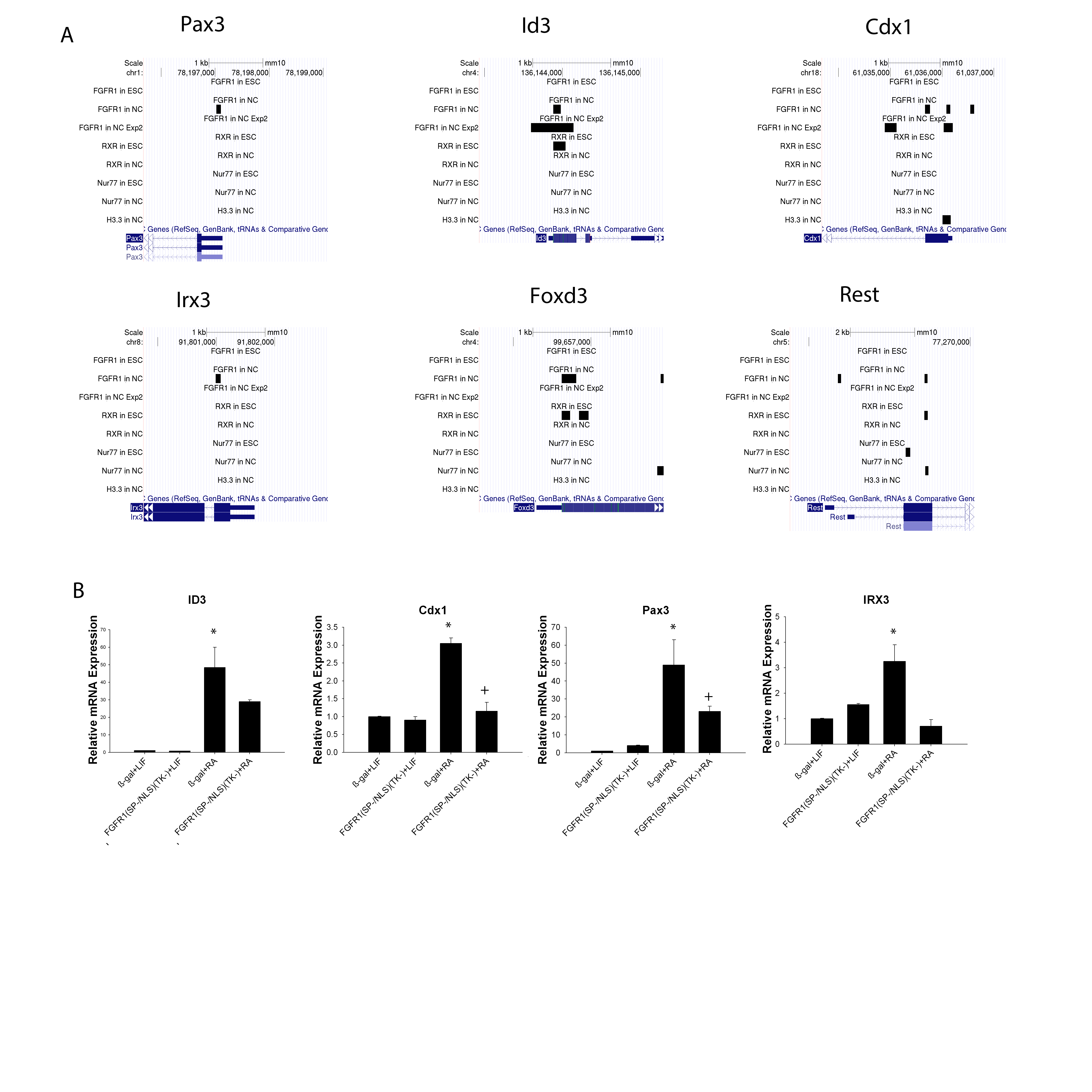

Supplement: S10 Fig — (A) UCSC genome browser views of nFGFR1, RXR and Nur77 binding within the promoter of Pax3, Id3, Cdx1 and Irx3. (B) FGFR1(SP-/NLS) (TK-) antogonizes or blocks the RA-induced activation of Pax3, Id3, Cdx1 and Irx3. mRNA expression levels were mesured using extracts from ESCs transfected with either β-gal (control) or FGFR1(SP-/NLS) (TK-) and subsequently maintained in the presence of +LIF or +RA for 48 hours. P value <0.05 * different from β-gal+LIF; + different from β-gal+RA. (TIFF) (TIF) [file pone.0123380.s010.tif]

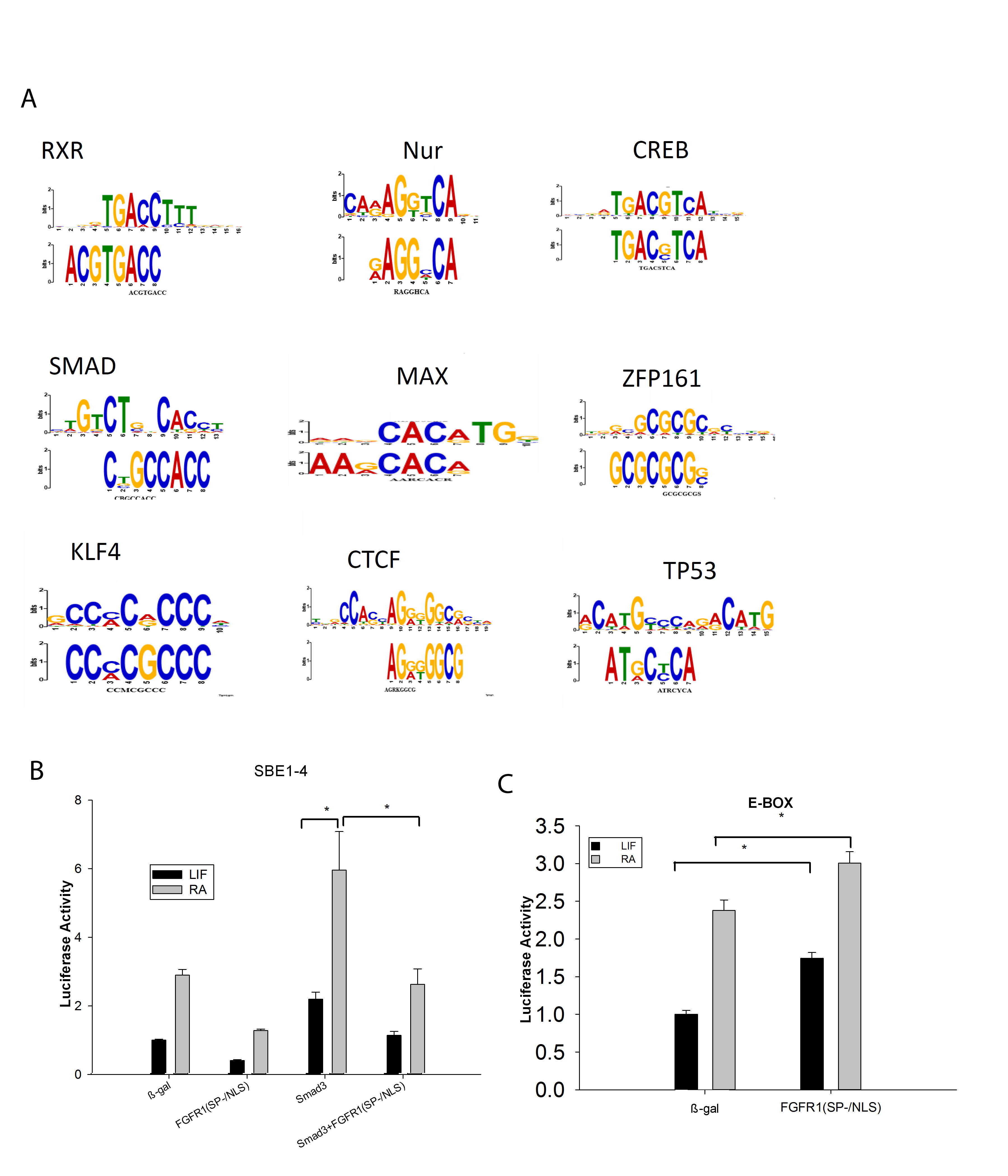

Supplement: S11 Fig — (A) All motif analyses were carried out using MEME-ChIP software. The over-represented DNA sequences reveal nFGFR1 binding to over 30 motifs representing consensus sequences of diverse TFs, all of which are known to interact with nFGFR1 binding partner and transcriptional coactivator CBP. Consistent with partial overlap of nFGFR1 peaks with RXR and/or Nur77, we found a number of the consensus sequences in which (i) nFGFR1 shares with RXR and/or Nur77 (ATF1,CTCF, MAX, NZF1, RXRα/Nur1, NRF1, RARα, RFX1, SMAD3, SOX8, SP1, STAT1, STAT3, YY1, ZFP161), (ii) consensus sequences targeted by nFGFR1 alone (ARNT, ERG/ELK4, KLF4, POU2F3, POU5F1/SOX2, SMAD2/4, TCF3, TP53, ZBTB33) and (iii) consensus sequences targeted by RXR and/or Nur77 but not shared with nFGFR1 (HIC1, IRX4, Mycn, PAX6,PITX2, POUF3, PPARG, PRRRX2, SIX6, ZEB1). In cases of TFs like CTCF, Klf4, SOX2, STAT3, and TP-53, nFGFR1 interacts both with their cognate DNA sequences as well as genes that encode these TFs. This implies a dual-level of regulation in which nFGFR1 controls generation of TFs as well as their downstream functions. (B) FGFR1(SP-/NLS) inhibits SBE-4 activation in the presence of Smad3. mESCs were transfected with the SBE-4-luc construct and either β-gal, FGFR1 (SP-/NLS), SMAD3 or SMAD3+FGFR1(SP-/NLS), and subsequently maintained in the presence of +LIF or +RA for 24 hours. Luciferase activity was normalized to β-gal. In the presence of RA, nuclear active FGFR1(SP-/NLS) significantly decreased the SMAD3-dependent activation of SBE4-luc. (C) FGFR1 (SP-/NLS) augments E-BOX activation in the presence of LIF and RA. mESCs were transfected with the E-BOX-luc construct and either β-gal or FGFR1(SP-/NLS), and subsequently maintained in the presence of +LIF or +RA for 24 hours. Luciferase activity was normalized to β-gal. Transfection of FGFR1 (SP-/NLS) significantly enhanced E-BOX dependent transcriptional activity in the presence of either LIF of RA treatment. (TIFF) (TIF) [file pone.0123380.s011.tif]
