# Supplementary material for: Global Developmental Gene Programing Involves a Nuclear Form of Fibroblast Growth Factor Receptor-1 (FGFR1)
Source: PLoS One. 2015 Apr 29;10(4):e0123380. doi: 10.1371/journal.pone.0123380 (PMC4414453; doi:10.1371/journal.pone.0123380)
Supplement: S4 Table — (DOCX) [file pone.0123380.s015.docx]

**S4 Table. Primers used in RT-qPCR assays.**

mRNA real-time RT-qPCR primers

| Hoxa1_RT_F-CCTGGCCACGTATAATAACTCC | Hoxa1_RT_R-GAAGTGGAACTCCTTCTCCAG |
| --- | --- |
| Hoxa2_RT_F-CTGCCTCGGCCACAAAGAATC | Hoxa2_RT_R-GCTTCATTCTCCGGTTCTG |
| Hoxa3_RT_F-  CCTGCTCAACTCTCCCACC | Hoxa3_RT_R-GCATAGGTAGCGGTTGAAGTG |
| Hoxa4_RT_F-  CTGGATGAAGAAGATCCAC | Hoxa4_RT_R-GGTTCTGAAACCAGATCTTG |
| Hoxa5_RT_F-  CCGAGGAGGACGCCCCTGC | Hoxa5_RT_R-GCGGGTCAGGTAGCGGTTG |
| Hoxa6_RT_F-CGGCAGCGTGCAGGGCAAAGC | Hoxa6_RT_R-GATGCGGCGCCGCCGAGTCAG |
| Hoxa7_RT_F-  CGCAGTTCAGGACCCGAC | Hoxa7_RT_R-GGCTGCAGTGGGAGCCTG |
| mKlf4_RT_F-GAAATTCGCCCGCTCCGATGA | mKlf4_RT_R-CTGTGTGTTTGCGGTAGTGCC |
| mE2f1_RT_F-CAGGGAAAGGTGTGAAATCTCCG | mE2f1_RT_R-GATGCCCTCCAGGACATTGGTGATG |
| CTCF_RT_F-ATGTGTGATTATGCCAGTGTAGAAGTCAG | CTCF_RT_R-CCACTCTGGGTAAACCGAGCGTGA |
| Nanog_RT_F-TGATTCAGAAGGGCTCAGCAC | Nanog_RT_R-CAGGTCTGGTTGTTCCAAGTTG |
| Oct4_RT_F-TTGGAAAGGTGTTCAGCCAGACCAC | Oct4_RT_R- ATCTCCTGAAGGTTCTCATTGTTGTC |
| Sox2_RT_F-TTACCTCTTCCTCCCACTCCAG | Sox2_RT_F-GCAGTGTGCCGTTAATGGC |
| Suz12_RT_F-AAAGGAAGGATGTAAGTTGTCC | Suz12_RT_R-CTTCTTCCTGGACGAGTTACT |
| Stat3_RT_F-  ACCCAACAGCCGCCGTAG | Stat3_RT_R-CAGACTGGTTGTTTCCATTCAGAT |
| Cyp26a1_RT_F-AAGGAGACCCTGCGATTGA | Cyp26a1_RT_R-GGATCTGGTATCCATTCAGCTC |
| Notch1_RT_F-GATTCATCTGTAGGTGCCCTGCG | Notch1_RT_R-GTTCTCGGATGTGGGCTCACAGGTG |
| Mesp2_RT_F-CCTGGCATGCAGATGTCCCCAGAG | Mesp2_RT_R-CTGAGGCTGAGGCTGAGGCTGAGG |
| Pax3_RT_F-  AAACCCAAGCAGGTGACAACG | Pax3_RT_R-  ATGCGGCTGATAGAACTCACTG |
| Id3_RT_F-  ACTACATCCTCGSCCTTCAGG | Id3_RT_R-  CTCCTCTTGTCCTTGGAGAT |
| Cdx1_RT_F-  AAGACCCGAACCAAGGACAAG | Cdx1_RT_R-  GCTGCTGCTGCTGTTTCTT |
| Irx3_RT_F-AAGAAGGAGAACAAGATGACG | Irx3_RT_R-  CGTCCGAGTCGCTAGTTTT |
