# Supplementary material for: Global Developmental Gene Programing Involves a Nuclear Form of Fibroblast Growth Factor Receptor-1 (FGFR1)
Source: PLoS One. 2015 Apr 29;10(4):e0123380. doi: 10.1371/journal.pone.0123380 (PMC4414453; doi:10.1371/journal.pone.0123380)
Supplement: S5 Table — (DOCX) [file pone.0123380.s016.docx]

**S5 Table. Primers used in ChIP-qPCR assays.**

ChIP-qPCR primers

| Hoxa1_ChIP_F-CCAGTGCTCCAAGTCAGC | Hoxa1_ChIP_R-CTGAGCCGCCTGCGAAAG |
| --- | --- |
| Hoxa2_ChIP_F-CATTGCCATCCACACCCACA | Hoxa2_ChIP_R-CACATGATTGCTTCTGCCAA |
| Hoxa4_ChIP_F- ACCCGAAGCCTGGTTGGACTG | Hoxa4_ChIP_R-CTTGGGTTCCGGCTGAGAAGTTG |
| Cyp26a1_ChIP_F-GCGGAACAAACGGTTAAAG | Cyp26a1_ChIP_R-CAGGTTACTGCCCACGTTA |
| Suz12_ChIP_F-CGCATCGTGTTGAGAAGCCAC | Suz12_ChIP_R-CATCTCCACTGAGGCAGGA |
| Mesp2_ChIP_F- CCTGGCATGCAGATGTCCCCAGAG | Mesp2_ChIP_R- CTGAGGCTGAGGCTGAGGCTGAGG |
